# Tumor-associated B-cells induce tumor heterogeneity and therapy resistance

**DOI:** 10.1038/s41467-017-00452-4

**Published:** 2017-09-19

**Authors:** Rajasekharan Somasundaram, Gao Zhang, Mizuho Fukunaga-Kalabis, Michela Perego, Clemens Krepler, Xiaowei Xu, Christine Wagner, Denitsa Hristova, Jie Zhang, Tian Tian, Zhi Wei, Qin Liu, Kanika Garg, Johannes Griss, Rufus Hards, Margarita Maurer, Christine Hafner, Marius Mayerhöfer, Georgios Karanikas, Ahmad Jalili, Verena Bauer-Pohl, Felix Weihsengruber, Klemens Rappersberger, Josef Koller, Roland Lang, Courtney Hudgens, Guo Chen, Michael Tetzlaff, Lawrence Wu, Dennie Tompers Frederick, Richard A. Scolyer, Georgina V. Long, Manashree Damle, Courtney Ellingsworth, Leon Grinman, Harry Choi, Brian J. Gavin, Margaret Dunagin, Arjun Raj, Nathalie Scholler, Laura Gross, Marilda Beqiri, Keiryn Bennett, Ian Watson, Helmut Schaider, Michael A. Davies, Jennifer Wargo, Brian J. Czerniecki, Lynn Schuchter, Dorothee Herlyn, Keith Flaherty, Meenhard Herlyn, Stephan N. Wagner

**Affiliations:** 1 The Wistar Institute, Philadelphia, PA 19104 USA; 2 Department of Pathology and Medicine, Perelman School of Medicine, University of Pennsylvania, Philadelphia, PA 19104 USA; 3 Division of Immunology, Allergy and Infectious Diseases (DIAID), Department of Dermatology, Medical University of Vienna, Vienna, A-1090 Austria; 4 New Jersey Institute of Technology, Newark, NJ 07102 USA; 5 Department of Dermatology and Venereology, Karl Landsteiner University of Health Sciences, St. Pölten, A-3100 Austria; 6 Department of Radiology, Division of Nuclear Medicine, Medical University of Vienna, Vienna, A-1090 Austria; 7 Department of Biomedical Imaging and Image-guided Therapy, Division of Nuclear Medicine, Medical University of Vienna, Vienna, A-1090 Austria; 8 Department of Dermatology and Venereology, The Rudolfstiftung Hospital, Teaching Hospital of the Medical University Vienna, Vienna, A-1030 Austria; 9 Department of Dermatology, Paracelsus Medical University Salzburg, Salzburg, A-5020 Austria; 10 Department of Pathology, The University of Texas MD Anderson Cancer Center, Houston, TX 77040 USA; 11 Department of Melanoma Medical Oncology, The University of Texas MD Anderson Cancer Center, Houston, TX 77040 USA; 12 Massachusetts General Hospital Cancer Center, Harvard Medical School, Boston, MA 02115 USA; 13 Melanoma Institute of Australia, and The University of Sydney, Sydney, 2065 Australia; 14 Department of Bioengineering, University of Pennsylvania, Philadelphia, PA 19104 USA; 15 Abramson Cancer Center, Hospital of University of Pennsylvania, Philadelphia, PA 19104 USA; 16 CeMM Research Center for Molecular Medicine of the Austrian Academy of Sciences, Vienna, A-1090 Austria; 17 Department of Biochemistry, McGill University, Montreal, QC Canada H3A0G4; 18 Dermatology Research Center, University of Queensland Diamantina Institute, The University of Queensland, Translational Research Institute, Brisbane, 4102 Australia; 19 Department of Surgical Oncology, The University of Texas MD Anderson Cancer, Center, Houston, TX 77040 USA; 20 Present Address: SRI International, Menlo Park, CA 94025 USA; 21 Present Address: Moffitt Cancer Center, Tampa, FL 33612 USA

## Abstract

In melanoma, therapies with inhibitors to oncogenic BRAF^V600E^ are highly effective but responses are often short-lived due to the emergence of drug-resistant tumor subpopulations. We describe here a mechanism of acquired drug resistance through the tumor microenvironment, which is mediated by human tumor-associated B cells. Human melanoma cells constitutively produce the growth factor FGF-2, which activates tumor-infiltrating B cells to produce the growth factor IGF-1. B-cell-derived IGF-1 is critical for resistance of melanomas to BRAF and MEK inhibitors due to emergence of heterogeneous subpopulations and activation of FGFR-3. Consistently, resistance of melanomas to BRAF and/or MEK inhibitors is associated with increased CD20 and IGF-1 transcript levels in tumors and IGF-1 expression in tumor-associated B cells. Furthermore, first clinical data from a pilot trial in therapy-resistant metastatic melanoma patients show anti-tumor activity through B-cell depletion by anti-CD20 antibody. Our findings establish a mechanism of acquired therapy resistance through tumor-associated B cells with important clinical implications.

## Abstract (web summary)

Resistance to BRAFV600E inhibitors often occurs in melanoma patients. Here, the authors describe a potential mechanism of acquired drug resistance mediated by tumor-associated B cells-derived IGF-1.

## Introduction

Melanoma is an aggressive form of skin cancer^1^.Advanced-stage melanomas are difficult to treat because tumors develop resistance to most therapies, including drugs targeting oncogenic BRAF^V600E[1]^. Also, only a third of melanoma patients show durable responses to immune checkpoint therapies^2^. Receptor-tyrosine kinase (RTK) mediated resistance to BRAF and BRAF/MEK therapy has been well described in in vitro models and patients’ tumor samples^1, 3–5^. However, the direct role of tumor stroma/microenvironment as the source of growth factors in therapy resistance has not been elucidated. In addition to the cancer cells, targeting infiltrating fibroblasts in the tumor microenvironment (TME) has been proposed as a novel treatment strategy for melanoma patients^3^. Our earlier studies suggest that an active interaction between melanoma cells and fibroblasts results in increased tumor growth and therapy resistance^6^. Besides fibroblasts, the tumor stroma includes immune cells such as neutrophils, macrophages, T cells and B cells^7^. Cross-talk between immune and malignant cells occurs either directly by cell–cell interactions or via soluble mediators such as growth factors and cytokines^7, 8^.

While the presence of melanoma-infiltrating T cells is associated with a favorable prognosis^9^, little information exists about the significance of tumor-infiltrating or tumor-associated B (TAB) cells, which represent up to ~33% of all infiltrating immune cells^9^. The frequency of TAB cells can be associated with improved prognosis in primary melanoma, but has also been associated with increased metastasis^9–11^ and shorter overall survival (OS)^12^. In murine melanoma models, the presence/level/activity of TAB cells correlates with increased angiogenesis and inflammation^13–15^, which is associated with STAT3 signaling in tumors and inflammatory cytokine production^13^. Evidence linking B cells to inflammation and malignant transformation has also come from squamous and pancreatic adenocarcinoma models, where chronic inflammation and malignant transformation was mediated through activation of myeloid or macrophage cells by immunoglobulins in the B-cell rich tissues^16–19^. In a prostate carcinogenesis model, B-cell-derived lymphotoxin promotes inflammation and transformation to castration-resistant carcinomas^20^.These compelling studies in mice and the prevalence of B cells in human melanoma and other cancers prompted us to examine their functional significance in metastatic melanoma.

In the present study, we investigated the cross-talk between B cells and tumor cells and determined whether and how this cross-talk can induce drug-resistance and associated tumor cell subpopulations. We further analyzed human tumor samples for the presence of identified mechanisms and, finally, evaluated B cells as therapy targets in a small clinical pilot trial in therapy-resistant metastatic melanoma patients. We uncover a critical mechanism of TAB-cell-mediated resistance to MAP kinase inhibitors with important clinical implications and highlight the role of the TME in modulating normal cells to enhance tumor cell survival.

## Results

### CD20^+^ B cells in tumor tissues and inflammatory cytokines

**Fig. 1:**
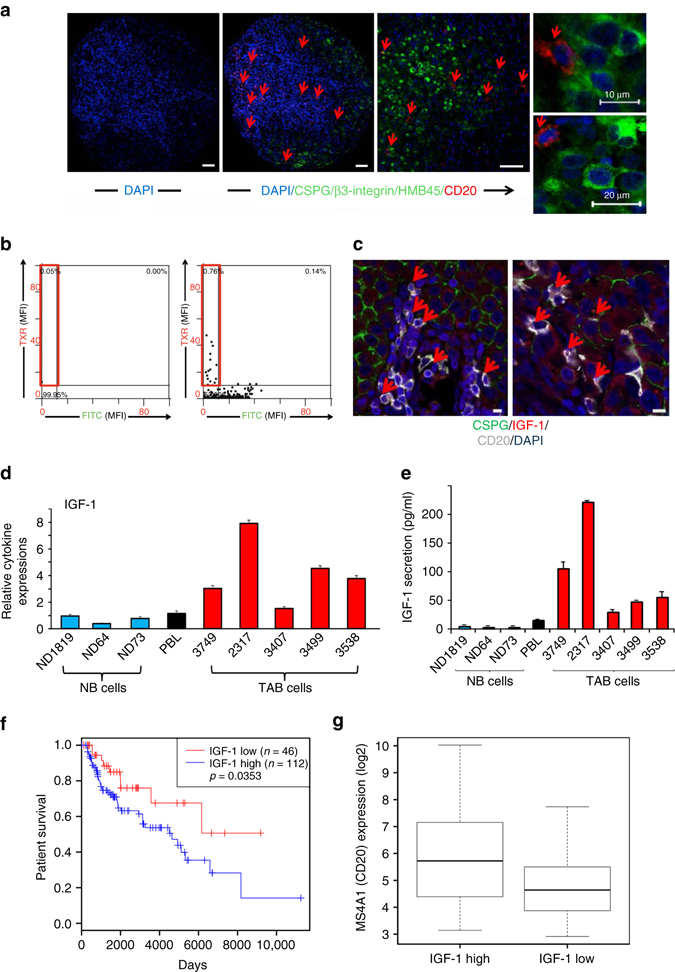
**a**–**g** Prevalence of CD20^+^ B cells in metastatic melanoma tissues and increased IGF-1 expression in TAB cells. **a**, **b** Presence of CD20^+^B cells. Representative immunostaining (TMA, 79 cores from 48 patients) for CD20 (*red*) and a three melanoma marker combination (CSPG/β3 integrin/HMB45 (*green*)) plus DAPI nuclear stain (*blue*). **a** *Left and middle panels*: sample with predominant distribution of CD20^+^, triple melanoma marker^-ve^ B cells in the tumor stroma (*red*; *left panel*: isotype control). *Right panel*: close-up with infiltration of single CD20^+^ B cells (*red*) among melanoma cells (*green*). *Scale bars*: 50 μm. *Far right 2 panels*: CD20^+^ B cells are DAPI^+^ and triple melanoma marker^-ve^. *Scale bars*: 10–20 μm. **b** Tissue FAXS analysis, the MFI was displayed in scattergrams for FITC-labeled CSPG/β3 integrin/HMB45+ and Texas red (TXR)-labeled CD20+ cells. Cutoff values were set based on isotype control staining (*left panel*). CD20+ B cells gated as CD20+, triple melanoma marker^-ve^cells (*right panel*, *red frame*). **c** Representative immunostainings from additional metastases (six patients’ biopsies) stained for IGF-1 (*red*) in CD20^+^ (*grayish white*) CSPG^-ve^ (*green*) B cells. *Scale bars*: 10 μm. **d**, **e** Immortalized B cells (48 h, B-cells-only cultures) from melanoma tissues (*n* = 5; TAB cells; *red bars*) show increased mRNA/protein expression of IGF-1 when compared with immortalized B cells from peripheral blood (*n* = 3; NB cells; *blue bars*) and pooled total PBL of healthy volunteers (*black bars*). **d** mRNA transcripts were determined by qPCR with levels indicated as RQ values normalized to GAPDH and relative to control normal B cells from healthy volunteers. *Bars* represent mean + SE of duplicate samples. Results are representative of three independent experiments for each sample. **e** B-cell supernatants (48 h, B-cells-only cultures) were used for protein expression of IGF-1 (ELISA). *Bars* represent mean + SE of duplicate samples. **f**, **g** TCGA-SKCM patients’ data set analyses (*n* = 473). **f** Kaplan–Meier survival curves of 158 melanoma patients with tumors containing a high lymphocyte infiltrate (above median, see Methods) who were divided into high and low IGF-1 expression (by median). Log-rank test shows poor OS in patients with high IGF-1 expression. **g** *Box plots* showing significantly (*p* < 0.0001, *t*-test) increased B-cell (MS4A1, CD20) expression levels in melanomas with high tumor IGF-1 expression

Quantitative cytometry of a tissue array from metastatic melanoma patients’ samples showed the presence of CD20^+^B cells (negative for melanoma-associated markers) in 17/48 lesions (33%; frequency of B cells (0.57%–28.8% of all cells); Fig. 1a, b) and biopsies from further six melanoma patients showed co-localization of IGF-1 in CD20^+^ B cells (Fig. 1c). We thus hypothesized that B cells within the TME support the malignant cells through expression of pro-inflammatory/pro-tumorigenic factors and cytokines.

We therefore collected normal B cells from peripheral blood of healthy volunteers and tumor-associated B cells from melanoma tissues (referred to hereafter as “fresh NB” and “fresh TAB” cells, respectively) and, due to their limited availability and shorter viability in vitro, immortalized some of these for use in longer-term assays (referred to hereafter as NB and TAB cells, see Methods). In contrast to normal immortalized and fresh NB cells from healthy individuals or melanoma patients, TAB cells produced IGF-1 (Fig. 1d, e), as did fresh TAB (Supplementary Fig. 1a), and a variety of other pro-tumorigenic/pro-inflammatory factors/cytokines including IL-1, VEGF, and PDGF (Supplementary Fig. 1b–g). Expression levels of IL-6 or TGF-β were unchanged between NB and TAB cells (not shown). To investigate the clinical significance of IGF-1 and TAB cells, we analyzed TCGA-SKCM melanoma data (*n* = 473) and observed a heterogeneous expression of B-cell signature genes (Supplementary Table 1; Supplementary Fig. 2a) and *IGF1* (Supplementary Fig. 2a) between tumor samples. Here, high IGF-1 levels in tumors with a high lymphocyte infiltrate (for clinical significance see Supplementary Fig. 2b) define a patient cohort with reduced OS (Fig. 1f) and are associated with increased *MS4A1* gene expression (Fig. 1g). In addition, expression levels of *IGF1* and *MS4A1* were correlated in stage IV (*r* = 0.5928 (*p* = 0.0199), Spearman’s rank correlation) melanomas.

We then focused on IGF-1 because it is not produced by melanoma cells but increases their survival^21^ and contributes to acquired resistance to BRAF inhibitors (BRAFi)^4^.

### Tumor B-cell cross-talk: induction of pro-tumorigenic factors

**Fig. 2:**
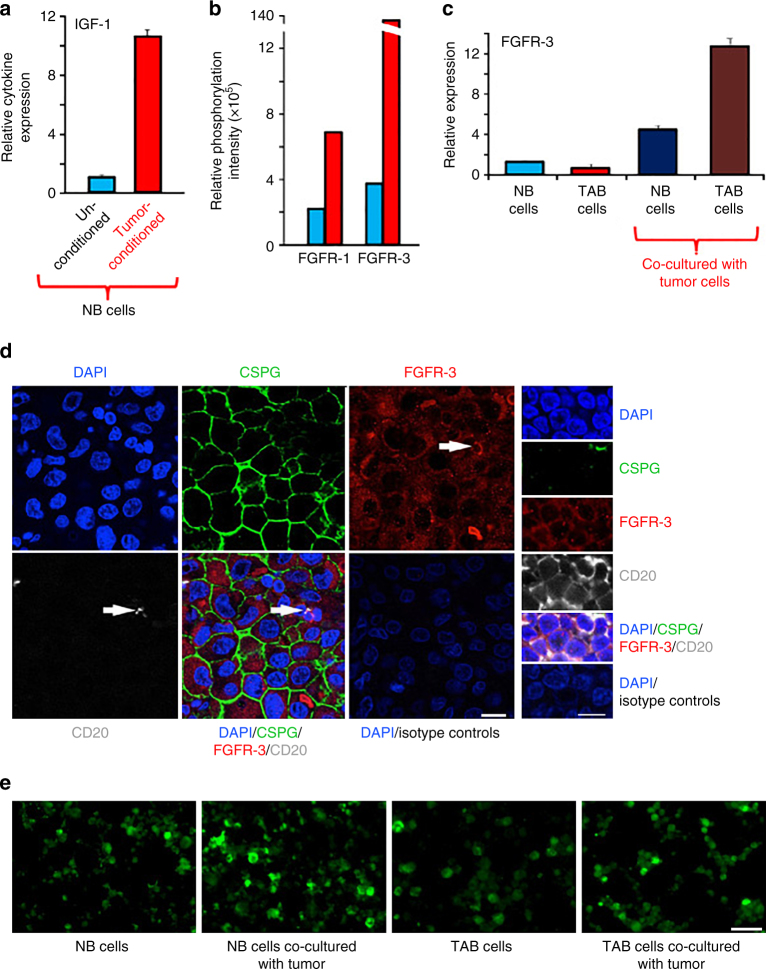
Melanomas convert normal B cells to a tumor-associated phenotype with high growth factor production for tumor stroma-tumor cell cross-talk. **a** NB cells from healthy volunteer (NB cells) co-cultured with melanoma tumor lineWM3749 (tumor-conditioned; *red bars*) show increased expression of growth factors and inflammatory cytokines, including IGF-1 (see Supplementary Fig. 4 for fresh NB cells), IL-1α/β and PDGF-A/-B (Supplementary Fig. 3b) when compared with unconditioned NB cells from the same healthy volunteer (*blue bars*). The B cells were harvested after 14 days (viability > 90%) and were analyzed by qPCR as in Fig. 1d. Results are representative of 2 independent experiments. **b**–**e** Upregulated protein or mRNA expression and phospho-signaling of FGFR-3 in B cells and melanoma cells as determined by qPCR or immunostaining. **b** NB cells co-cultured with melanoma cells for 48 h (*red bars*) show increased phosphorylation of FGFR-1 and FGFR-3 when compared with unconditioned B cells (*blue bars*) as determined by phospho-RTK array analysis. **c** NB- (*dark blue bar*) and TAB cells (*dark brown bar*) co-cultured with melanoma cells (WM3749) for 48 h show increased FGFR-3 expression compared with unconditioned normal (*blue bar*) or TAB cells (*red bar*); qPCR as in Fig. 1d. Results are representative of two independent experiments. **d** Detection of FGFR-3 expression in TAB cells in additional 10 metastatic melanoma patients’ biopsies by laser scanning microscopy. The 6 larger images on the left show FGFR3 expression in a CD20^+^ (*grayish white*) CSPG^-ve^ (*green*) B-cell (white arrow) as well as in melanoma cells (CSPG^+^; *green*). The right panel is a close up into a CD20+ (*grayish white*) lymphocyte cluster of the tumor stroma in direct apposition to melanoma cells (part of a melanoma cell can be seen in the upper right corner by staining for CSPG; *green*). Nuclei were counterstained with DAPI (*blue*). Isotype-matched antibodies were used as a control. Representative images are shown. *Scale bars*: left 25 μm, right 10 μm. **e** Detection of FGFR-3 expression in NB or TAB cells after co-culture (48 h) with melanoma cells (WM3749) detected by immunostaining. Cytospin preparation of normal B or TAB cells after co-culture with melanoma cells show upregulation of FGFR-3 as determined by staining of B cells with rabbit anti-FGR-3 antibody followed by Alexa-Fluor 488 conjugated anti-rabbit antibody. *Scale bars*: 40 μm. Images were captured using Nikon fluorescent microscope

We first sought to determine whether melanoma cells send signals to B cells to acquire a tumor-supportive phenotype, which is characterized by the expression of diverse pro-inflammatory/pro-tumorigenic factors and cytokines. To this end, a co-culture system was established where tumor and normal B cells were separated by a semi-permeable membrane (0.4 μM) that allows only the exchange of soluble mediators (Supplementary Fig. 3a). After 14 days, (tumor-conditioned) NB cells showed high transcript levels for IGF-1 (Fig. 2a), IL-1 (α/β) and PDGF-Α/-Β (Supplementary Fig. 3b) whereas non-co-cultured (unconditioned) NB cells expressed low levels or none at all. Tumor-conditioned fresh NB cells showed a similar increase in IGF-1 (Supplementary Fig. 4).

To identify receptor/ligand pair interactions, a RTK-phospho-array was performed using cell lysates of tumor-conditioned B cells (48 h co-culture). Moderate to high phosphorylation of FGFR-1/FGFR-3 (Fig. 2b; Supplementary Fig. 5a, b), S6 ribosomal protein and STAT3 (Supplementary Fig. 5a, b) was induced in co-cultured NB and TAB cells and accompanied by induction of FGFR-3 transcript (Fig. 2c) and protein expression (by immunofluorescence, Fig. 2e). Consistently, a pronounced expression of FGFR-3 on B cells and on tumor cells could be seen in metastatic melanoma samples (Fig. 2d). FGF-2 produced by melanoma cells is a natural ligand for FGFR-3.

**Fig. 3:**
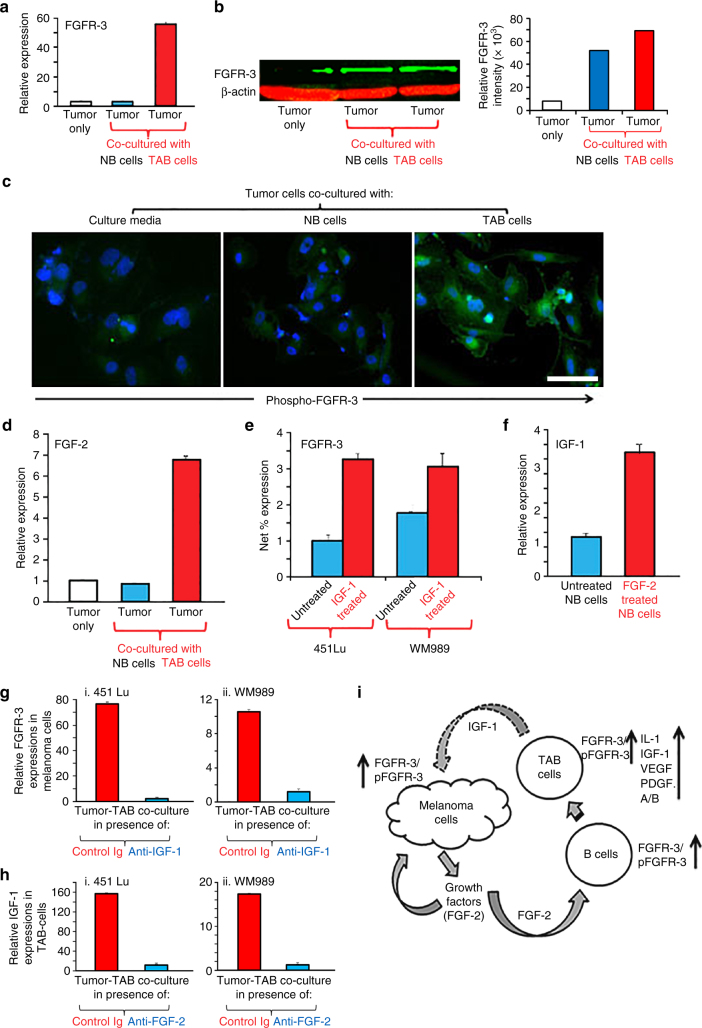
TAB cells modulate melanoma cells to express FGFR-3 and its ligand FGF-2 for tumor stroma-tumor cell cross-talk. **a** WM3749 co-cultured (72 h) with TAB cells (*red bar*) show increased FGFR-3 mRNA expression when compared with tumors only (*open bar*) or co-cultured with NB cells (*blue bar*). **b** Lysates obtained from pools of melanomas co-cultured (72–120 h) with NB- or TAB cells were probed in western blot with anti-FGFR-3 antibody (*left panel*), results expressed as relative intensity after β-actin normalization (*right panel*). **c** Melanoma cells co-cultured with TAB cells (72 h) show increased phospho-FGFR-3 expression (*right panel*; immunofluorescence assays) when compared with melanoma cells alone (*left panel*) or melanoma cells co-cultured with NB cells (*middle panel*), *scale bars*: 40 μm, images captured by Nikon inverted microscope. **d** Melanoma cells co-cultured with TAB cells (*red bar*) show increased FGF-2 mRNA expression when compared with melanoma cells alone (*open bar*) or melanoma cells co-cultured with NB cells (*blue bar*). **e** 451Lu and WM989treated with IGF-1 (25 ng/ml/daily for 5 days; *red bar*) show increased FGFR-3 expression when compared with untreated controls (*blue bar*), flow cytometry results expressed as net % expression of control antibody. IGF-1 treated melanoma cells (*red bars*) show higher expression of FGFR-3 compared with untreated cells (*blue bars*). Bar represents mean + SD of replicate samples. **f** NB cells treated with FGF-2 (10 ng/ml/daily for 4 days; *red bar*) show high IGF-1 mRNA expression relative to untreated NB cells (*blue bar*). **g** 451Lu and WM989 co-cultured (72 h) with TAB cells in the presence of an anti-IGF-1 neutralizing antibody (10 μg/ml) show decreased FGFR-3 mRNA expression in tumor cells (*blue bar*) when compared with controls (*red bar*). **h** TAB cells co-cultured (72 h) with 451Lu and WM989 in the presence of an anti-FGF-2 neutralizing antibody (1 μg/ml) show decreased IGF-1 mRNA expression in B cells (*blue bar*) when compared with controls (*red bar*).Experiments in **a**, **d** and **f**–**h** were performed using qPCR. In Figures **a**, **d**–**h**, bars represent mean + SE of duplicate samples and are representative of at least two independent experiments. **i** Summary of cross-talk between melanoma and B cells: FGF-2 is constitutively expressed by tumor cells, released into the microenvironment to bind FGFR-3 on the B cells, activated B cells express increased levels of pro-inflammatory cytokines. IGF-1 released by TAB cells modulates tumor cells to increase their growth, heterogeneity and therapy resistance

We next investigated whether co-cultured tumor cells respond to B cells and their secreted factors. TAB cells, in contrast to NB cells, stimulated FGFR-3 expression (by qPCR, Fig. 3a; single-cell FGFR3 mRNA FISH, Supplementary Fig. 6; western blotting, Fig. 3b) and activation (by immunofluorescence, Fig. 3c) in tumor cells and increased the expression of FGF-2 (Fig. 3d). Induction of FGF-2 and FGFR-3 expressions in melanoma cells could be detected from 48 h on.

Recombinant IGF-1 increased tumor cell expression of FGFR-3 (Fig. 3e) as did co-cultured TAB cells, and reciprocally, recombinant FGF-2 induced IGF-1 production in NB cells (Fig. 3f) as did co-cultured tumor cells (Fig. 2a). Induction of IGF-1 expression in B cells could be detected from 48 h on. Consistently, neutralization of IGF-1 in TAB-tumor cell co-cultures led to inhibition of FGFR-3 induction (Fig. 3g) and FGF-2 secretion (Supplementary Fig. 7a) by tumor cells. Also, neutralization of FGF-2 in co-cultures showed inhibition of IGF-1 induction (Fig. 3h) and secretion (Supplementary Fig. 7b) by B cells.

Together, these data support a FGF-2-induced conversion of B cells into a tumor-supportive phenotype which, with IGF-1 as critical key growth factor, reciprocally provides sustained pro-inflammatory and pro-tumorigenic signals to melanoma cells leading to activation of FGFR-3 (Fig. 3i).

### Heterogeneous subpopulation induction by TAB cells and IGF-1

**Fig. 4:**
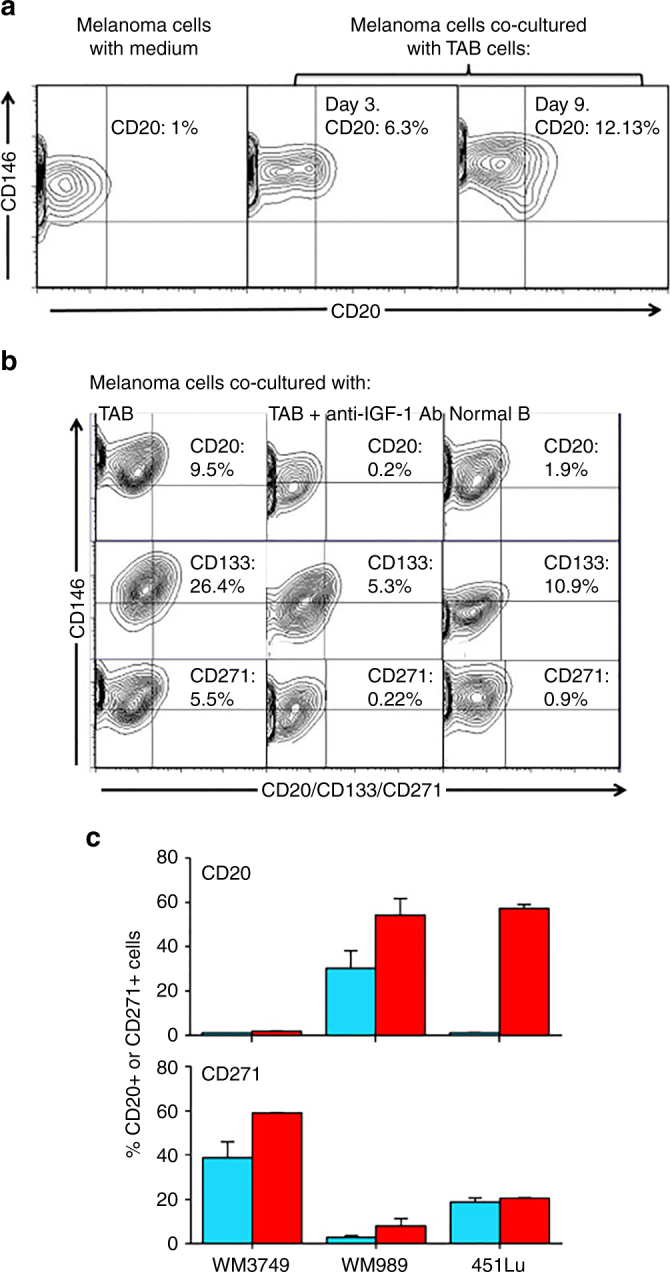
IGF-1-dependent induction of cancer stem cell markers CD20, CD133, and CD271 (NGFR) on melanoma cells. **a** Melanoma cells (WM3749) co-cultured with TAB cells (days 3 and 9) show high expression of CD20 (*middle and right panels*) compared with the control culture (*left panel*) as determined by FACS analysis. Melanoma cells were co-stained with anti-CD146 (MCAM, PE-conjugated) and anti-CD20 (FITC-conjugated) antibodies to distinguish them from B cells, which are CD146-negative;percentages indicate co-expression of both markers on the malignant cells. **b** Melanoma cells (WM3749) co-cultured with TAB cells (day 6) show high expression of CD20, CD133 and CD271 (*left panel*) compared with minimal or low expression of those markers when tumor cells are co-cultured with NB cells (*right panel*). Co-culture of melanoma cells with TAB cells did not modulate the expression of CD144 (vascular-endothelial cadherin marker) that are normally expressed by aggressive melanomas (data not shown). Induction of CD20, CD133, and CD271 was blocked when anti-IGF-1 neutralizing antibody (10 μg/ml) was used in the co-culture (*middle panel*). Anti-IL-1, anti-PDGF or anti-VEGF antibodies had no effect on CD marker expression (data not shown). Percentages indicate co-expression of CD20, CD133, or CD271on CD146^+^ melanoma cells. Results are representative of two independent experiments. **c** Melanoma cells(WM3749, WM989 and 451Lu) cultured in the presence of recombinant IGF-1 (25 ng/ml) for 5 days showed high expression of CD20 or CD271 (*red bars*) compared with untreated cells (*blue bars*) as determined by FACS assay. *Bar* represents mean + SE of triplicate samples

The cross-talk between tumor cells and the TME can contribute to melanoma heterogeneity through the induction of distinct tumor-supportive subpopulations^22^. We therefore examined the potential of TAB cells to induce melanoma subpopulations in our co-culture system (Supplementary Fig. 3a). Tumor cells co-cultured with TAB cells (days 3–9) showed the induction of a CD20^+^ subpopulation as indicated by immunophenotyping (Fig. 4a). Induction of CD20 on tumor cells was blocked by a CD20 shRNA (Supplementary Fig. 8a, b). CD20^+^ melanoma cells have cancer stem cell-like activities^23, 24^. TAB cells further induced CD133^+^ and CD271^+^ subpopulations (Fig. 4b), which also have a cancer stem cell-like phenotype and are linked to therapy resistance^24^. The induction of CD271 was additionally confirmed by live-cell imaging of melanoma cells transduced with a CD271-dsRed promoter construct (Supplementary Fig. 8c). Induction of the subpopulations was seen in most melanoma cells tested (Fig. 4c) and was blocked by antibodies to IGF-1 (Fig. 4b) but not by a control isotype antibody (Supplementary Fig. 8d).

The important role of the cross-talk between tumors and B cells in the induction of melanoma subpopulations was further supported when TAB cells where substituted with tumor-conditioned or un-conditioned NB cells (Supplementary Fig. 8e). Only tumor-conditioned NB cells could induce the CD20^+^ cancer phenotype as TAB cells did (Fig. 4a).

### Induction of therapy resistance by TAB cells and IGF-1

**Fig. 5:**
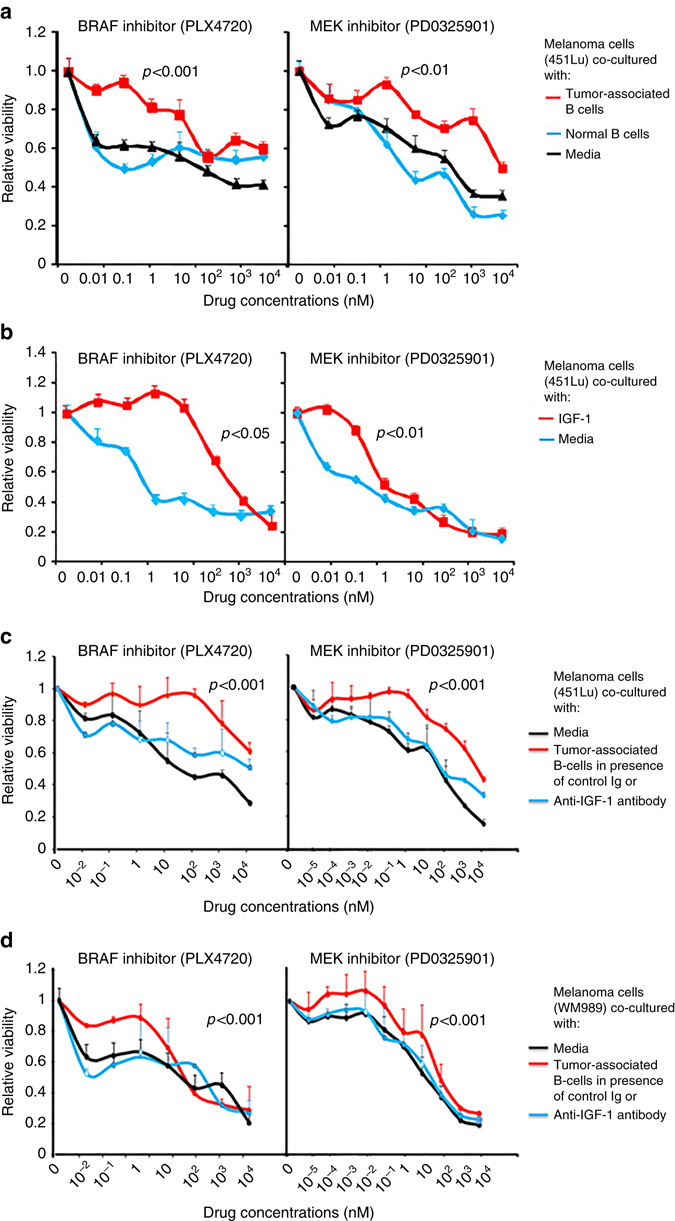
Melanoma cells co-cultured with TAB cells or recombinant IGF-1 are resistant to signaling inhibitors. **a** Melanoma cells (451Lu) co-cultured with TAB cells for 4–5 days are resistant to subsequent BRAFi (PLX4720) and MEKi (PD0325901) treatment (*red lines*) as compared with tumor cells cultured in the presence of NB cells (*blue lines*) or media control only (*black lines*). Co-cultured tumor cells were treated with drugs in triplicates for 72 h and viability was determined using the AlamarBlue assay. Results are expressed as relative viability of melanoma cells and drug responses are compared with appropriate controls. Data represents mean + SE of triplicate samples and *p* values (*t*-test) as indicated are for drug doses 0.1 to 10 nM of BRAFi and 0.01 nM to 1 nM of MEKi. **b** Melanoma cells treated with recombinant IGF-1 show resistance to BRAFiand MEKi. Melanoma cells (451Lu) were treated every day for a total of 5 days with recombinant IGF-1 (25 ng/ml; *red lines*), then harvested and their dose responses to BRAFiand MEKi determined as above. Results are compared with untreated melanoma cells as controls (*blue lines*). **c**, **d** Melanoma cells (451Lu (**c**) and WM989 (**d**)) co-cultured with TAB cells for 4 days in presence of anti-IGF-1 neutralizing antibody (*blue lines*) or media control (*black lines*) are sensitive to subsequent BRAFi and MEKi treatment as compared with cultures incubated with control antibody (*red lines*)

Melanomas undergoing treatment with BRAFi and MEKi invariably show acquired resistance to therapy drugs. Since normal host cells cross-talk with tumors we asked whether TAB cells confer drug resistance to melanoma cells. While tumor cells showed a dose-dependent reduction in cell viability to BRAFi and MEKi when co-cultured with NB cells, they showed significant resistance to both inhibitors (Fig. 5a) and chemotherapeutics (cisplatin or paclitaxel; not shown) upon co-culture with TAB cells.

**Fig. 6:**
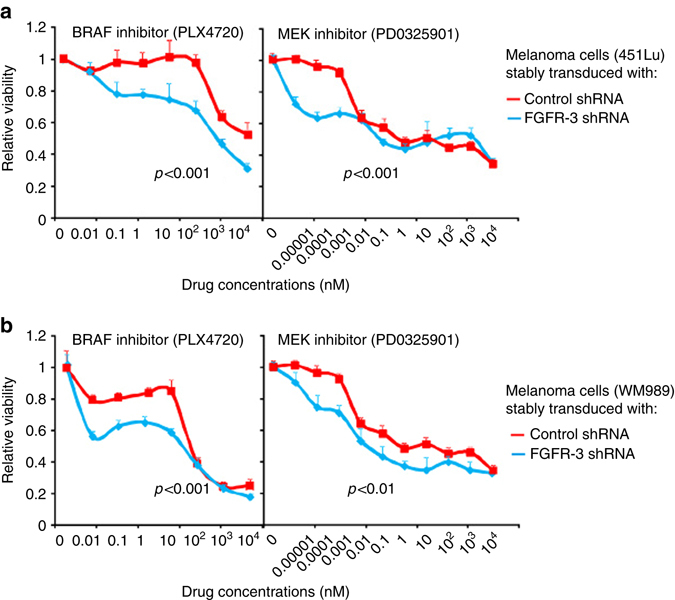
Knock-down of FGFR-3 in melanoma cells restores sensitivity to signaling inhibitors. **a**, **b** Knock-down of FGFR-3 rescues the sensitivity to BRAFi and MEKi. Dose responses to BRAFi and MEKi of melanoma cell lines (451Lu (**a**) and WM989 (**b**) stably transduced with FGFR-3 shRNA (*blue lines*) were determined as in Fig. 5 and compared with control shRNA-transduction (*red lines*). For validation of the FGFR-3 shRNA clone see Supplementary Fig. 10

As did TAB cells, recombinant IGF-1 significantly preserved the viability of melanoma cells against BRAFi and MEKi (Fig. 5b) further supporting its role as a tumor-supportive TAB-cell phenotype-defining mediator. Consistently, neutralization of IGF-1 in TAB and tumor cells co-culture rescued the sensitivity of melanoma cells to BRAFi and MEKi (Fig. 5c, d). We then asked whether tumor cells treated with the BRAFi PLX4720 can induce IGF-1 in B cells as did untreated melanoma cells (Fig. 2a). Pretreatment with BRAFi did not change the ability of 2 of 3 tested melanoma cell lines to induce IGF-1 expression in NB cells; one cell line lost this ability presumably due to modulation of FGF-2 activity (Supplementary Fig. 9). Further, in line with our observation that TAB cells induce drug resistance via IGF-1 (Fig. 5c, d) and activation of the FGFR-3 receptor in tumor cells (Fig. 2b; Fig. 3a, c, e, g), drug resistance of melanoma cells was dependent on the presence of FGFR-3. After shRNA interference with FGFR-3 expression (Supplementary Fig. 10a, b), tumor cells lost their resistance against BRAFi and MEKi in co-cultures with TAB cells (Fig. 6a, b).

To find additional clinical evidence for in vitro findings, we next analyzed two independent melanoma patient cohorts where tumor DNA/RNA material and tissue sections were available together with treatment response data to BRAFi mono- and BRAFi/MEKi combination therapies (Supplementary Tables 2 and 3).

**Fig. 7:**
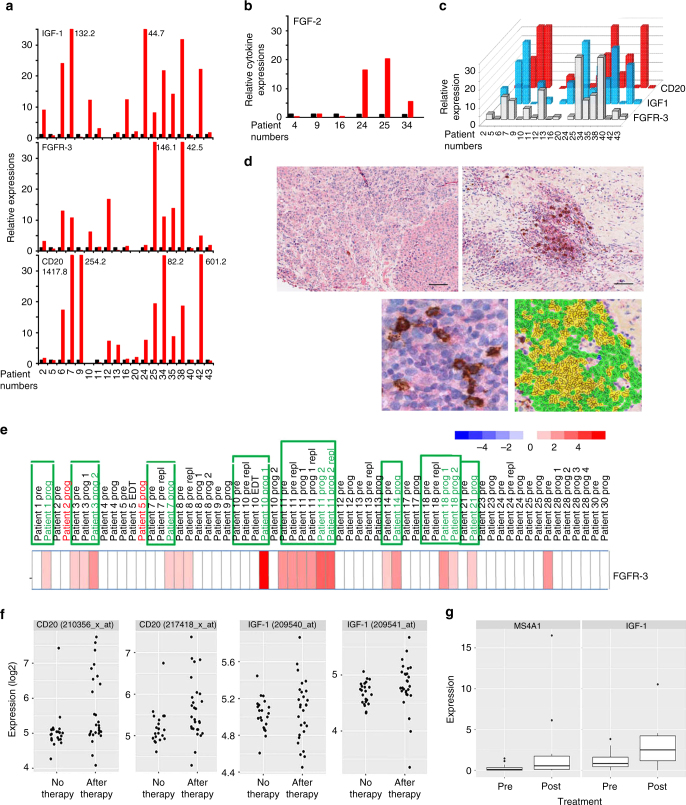
Increased expression of IGF-1, FGFR-3, its ligand FGF-2 and CD20 in tumor tissue obtained on treatment with BRAF and MEK inhibitors. **a**, **b** Tumor tissue from melanoma patients (*n* = 20) after kinase inhibitor therapies (*red bars*) show increased transcript levels of IGF-1, FGFR-3, CD20, and FGF-2 **b** when compared with same patients’ pre-treatment tumor tissues (*black bars*). mRNA transcripts were determined by real time qPCR (as described in Fig. 1 legend) with levels indicated as RQ values normalized to an endogenous control (GAPDH) and relative to pre-treatment cDNA samples. **c** Transcript levels of IGF-1, FGFR-3, and CD20 of melanoma patients’ cDNA samples on treatment showed a correlation with each other (IGF-1 and FGFR-3 (Spearman’s *r* = 0.6936; *p* = 0.0376); IGF-1 and CD20 (Spearman’s *r* = 0.5074; *p* = 0.0020)). **d** Increased presence of CD20^+^ B cells co-stained with IGF-1 in tumor sections obtained from patients undergoing treatment with kinase inhibitors. Representative immunostaining of a patient’s tumor section pre- (*left top panel*) and on-therapy (*bottom left panel*) with BRAFi/MEKi showing co-staining of IGF-1 (*red*) and CD20 (*dark brown*). Magnified view of the co-staining is shown on top right panel and multi-spectral analysis confirming the co-localization of IGF-1 and CD20+ B cells is shown in bottom right panel (*yellow*). *Scale bars*: 100 μm. **e** Increased RNA expression of FGFR-3 in 8/21 progression biopsies (*green frame*) obtained post BRAF inhibitor (dabrafenib) therapy when compared with pretreatment biopsies. Gene expression analysis was performed using GEO data set (GSE50509). **f** Gene expression analysis of GEO data set (GSE8401, *n* = 52 metastatic melanoma samples): scatter plots showing significantly higher CD20 RNA expression (two probes; *left two panels*; *p* = 0.0185 and *p* = 0.0125; *t*-test) (see also Supplementary Fig. 11) and a trend towards higher IGF-1 expression (two probes; *right two panels*) in therapy resistant samples. **g** RNA-seq data were downloaded from EGA under accession number EGAS00001000992 (*n* = 38 melanoma samples, 27 patients) and the data from multiple probe sets are summarized into scatter plots. Significantly higher CD20 (MS4A1; *left panel*; *p* = 0.0206) and IGF-1 RNA expression (*right panel*; *p* = 0.0403; *t*-test) are seen in BRAFi-resistant melanomas

*In the first cohort*: we compared transcript levels from matched pre-treatment and BRAFi mono- or BRAFi/MEKi combination therapy-resistant tumor samples (*n* = 20; Supplementary Table 2; from Massachusetts General Hospital Cancer Center). In line with our previous results on the induction of therapy resistance by B cells via IGF-1 secretion (Fig. 5a–d), we observed increased CD20 levels in therapy-resistant vs. pre-treatment samples in 11/19 matched pairs (58%; 95% confidence interval (34–78%))and a correlation of CD20 with IGF-1 transcript levels ((*r* = 0.5074, Spearman’s rank correlation); Fig. 7a, c).

FGF-2 expression was studied in fewer matched pairs (*n* = 6) due to limited material and 3/6 showed increased expression in treatment-resistant vs. pre-treatment samples (Fig. 7b), again together with increased levels of CD20 transcripts (samples 24, 25, 34; Fig. 7a).

Expression of IGF-1 in CD20^+^/PAX5^+^ B cells could be demonstrated by immunostaining of biopsies obtained from eight patients on treatment with BRAFi/MEKi combination(Fig. 7d; Supplementary Fig. 11a–c). Again, we observed an increase from 0.11% of IGF-1^+^/CD20^+^/PAX5^+^ B cells (percent of all cells) in pre-treatment to 1.2% in on-treatment biopsies.

A role for FGFR-3 in therapy resistance was supported by increased FGFR-3 transcript levels in 10/19 (53%; 95% confidence interval (31–76%)) matched BRAFi/MEKi treatment-resistant vs. pre-treatment samples and correlated well with IGF-1 transcript levels ((*r* = 0.6936, Spearman’s rank correlation); Fig. 7a, c).

*In a second independent cohort*: a gene expression analysis performed on pre-treatment and dabrafenib-resistant biopsies from 21 patients further supported the role of FGFR-3 in therapy resistance^5^. Of these, eight patients (~38%) showed increased FGFR-3 transcript levels in matched treatment-resistant samples as compared with pre-treatment samples (Fig. 7e) and almost all (7/8) showed the presence of CD20^+^TAB cells by immunostaining (Supplementary Table 3).

To further support the role of tumor-infiltrating CD20^+^ B cells in therapy resistance, we compared gene expression array (GSE8401, *n* = 52) and RNA-seq data (EGAS00001000992, *n* = 38) from further two independent cohorts of metastatic pre-treatment and treatment-resistant melanoma samples (Supplementary Fig. 11d; Fig. 7f, g). Expression array data showed a subgroup of tumor samples clustered by high CD20/CD19 expression enriched for post-treatment samples resistant to various therapies (Supplementary Fig. 11d) and a significant increase for two CD20 probes in post- as compared with pre-treatment samples (Fig. 7f, *2 left panels*). RNA-seq data of patients treated with a BRAFi showed a significantly higher CD20 expression in on-/post-treatment as compared with pre-therapy biopsies (Fig. 7g, *left panel*). In these data, a similar significant increase in IGF-1 expression was observed (Fig. 7g, *right panel*) and a trend towards an increase in expression array data (Fig. 7g, *2 right panels*).

### A pilot trial of CD20 immunotargeting in melanoma patients

The clinical relevance of our data was further highlighted in a small, prospective, multicenter pilot trial on B-cell depletion with anti-CD20 antibody ofatumumab in patients with therapy-resistant advanced metastatic melanoma (see Methods, Supplementary Fig. 12; NCT01376713). We included 10 patients with disease progression under kinase and/or checkpoint inhibitors or, who could not be considered for those therapies, other treatments. At study entry, all patients had disease progression and a baseline level of lactate dehydrogenase (LDH) above the upper limit of the normal range, most had extensive stage IV disease (80% visceral metastasis, 70% ≥ 4 metastatic body sites) and the majority (70%) had at least 2 to 5 systemic therapies before (Supplementary Table 4). Together, this study collective represented a heavily pretreated, end-stage, very poor prognosis group of melanoma patients.

**Fig. 8:**
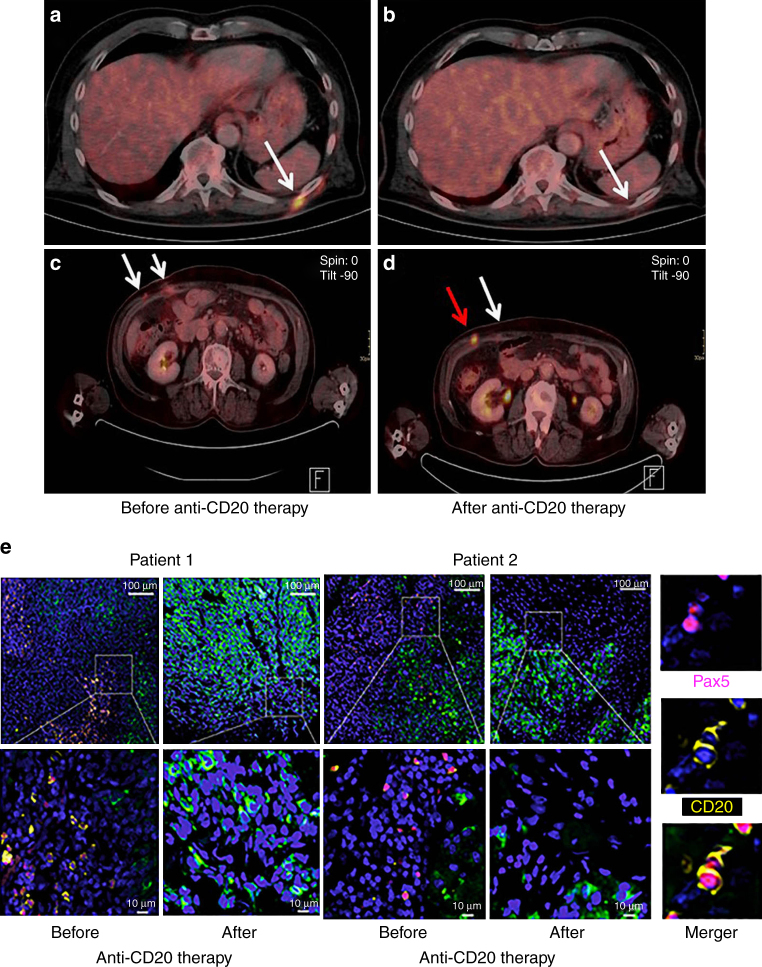
Clinical activity of CD20 immunotargeting in metastatic melanoma patients. PET-CT scans from two different patients obtained pre- vs. post-anti-CD20 antibody therapy. **a**, **b** Note complete disappearance of a metabolically active metastatic site (*white arrow*); **c**, **d** mixed response with almost complete disappearance of one metabolically active metastatic site (*white arrow* in **d**) and simultaneous increase in size and metabolic activity of the other (*red arrow* in **d**). **e** Representative combined PAX5 (nuclear; *purple*)/CD20 (membrane; *yellow*) immunofluoresence staining of patient-matched melanoma samples before and after therapy with anti-CD20 antibody (overviews (*top rows*; *scale bars*: 100 μm) and corresponding close ups (*bottom rows*; *scale bars*: 10 μm)). Note depletion of TAB cells in post-therapy tumors. CSPG staining of melanoma cells (*green*) and nuclear DAPI staining (*blue*). *Right panel*: double positive PAX5 (nuclear)/CD20 (membrane) immunofluoresent TAB cells

Evidence for clinical activity of B-cell depletion (by RECIST v1.1^25^ and ir-RC criteria^26^, mixed responses, induction of necrotic tumor masses or a reduction of standardized uptake values (SUVs) in FDG-PET-CT scans) was observed in 8 of 10 patients (Fig. 8a–d; Supplementary Table 5); post-therapy tumor samples showed depletion of TAB cells (Fig. 8e).

A benefit in measurable disease (primary end point: disease control rate, intention to treat analysis) as assessed by both RECIST and ir-RC criteria (Supplementary Table 5) was seen in 6 patients. One patient achieved a confirmed partial response (PR) with a more than 35% decrease of measurable disease, and further five patients showed a stable disease (SD, 4 with decrease of measurable disease), which lasted from 8 to 22+ weeks; patients then moved to rescue arm or ended trial. Even two of the four patients with progression (PD) of measurable disease showed signs of clinical activity such as induction of necrotic tumor masses or reduction of SUVs. In non-target lesions, B-cell depletion resulted in mixed responses in half of the patients (*n* = 5) with sometimes complete regression (CR) of single tumor lesions and simultaneous appearance of new lesions (mixed responses, Fig. 8c, d).

Overall response evaluation of the pre-specified primary end point of this trial, disease control rate (CR, PR or stable disease (SD) > 4 months), showed SD in two patients by ir-RC and in 1 patient by RECISTv1.1 criteria, and PD in other patients.

## Discussion

Our study demonstrates a mechanism of drug resistance in melanoma that is conferred by B cells from the TME. FGF-2 produced constitutively by tumors induces B cells to produce inflammatory factors and cytokines, most notably IGF-1. The resulting cross-talk leads to melanoma heterogeneity and resistance to kinase inhibitors.

Unlike T cells, the role of B cells in cancer progression is largely unknown. This is despite the presence of high infiltrating B cells in many human cancers (breast, colon, lung, prostate, ovary, and skin (melanoma))^9, 27–32^. The few available reports provide mixed views about the role of TAB cells and disease progression^9, 10, 12, 17, 28–30, 32^.

In mouse squamous cell carcinoma, a regulatory B-cell subset is involved in the transformation of premalignant lesions and depletion of B cells resulted in improved responses to chemotherapy^17^; in murine pancreatic ductal adenocarcinoma, progression of pancreatic neoplasia is linked to increased infiltration of immunoglobulin or IL-35 secreting B cells^18, 19, 33^; and in murine castration-resistant prostate cancer, IL-10, and PD-L1 expressing B cells impede T-cell-dependent chemotherapy responses^34^. In humans, there are no well-defined markers for B-cell subsets^35^. Therefore, we have delineated B cells based on their cytokine profiles. Both tumor-associated and normal B cells conditioned by melanoma cells express high levels of inflammatory cytokines (excluding VEGF; Fig. 2a; Supplementary Figs. 3 and 4). The clinical relevance of these in vitro data is supported by IGF-1 and CD20 co-expression in melanoma biopsies (Fig. 1c). Also, TCGA data set analyses (Supplementary Fig. 2a, b) confirm the co-expression of IGF-1 with B-cell genes in melanoma biopsies and higher IGF-1 level to be associated with an enhanced frequency of TAB cells (Fig. 1g). This suggests that increased intratumoral levels of IGF-1 may counter (Fig. 1f) the favorable effects of tumor-lymphocyte infiltrates on patients’ OS (Supplementary Fig. 2b).

FGF-2 produced by melanoma cells has a key role in the conversion of normal B to TAB cells. The cross-talk with TAB cells leads to enhanced FGF-2 and FGFR-3 expression in tumors resulting in a pro-survival phenotype. In our study, two independent sets of clinical data provide evidence for increased FGFR-3 expression in biopsies resistant to BRAFi alone or BRAFi and MEKi combination therapies (Fig. 7a, e). FGF-2/FGFR-3 signaling is involved in angiogenesis, tumor progression, and therapy resistance in other cancers^36^.

Our data show that TAB cells modulated tumor functions in two ways: (i) by inducing heterogeneous subpopulations, and (ii) by conferring therapy resistance. IGF-1 released by TAB cells induces heterogeneous tumor subpopulations with cancer stem cell-like characteristics that are defined as CD20^+^, CD133^+^, or CD271^+^. Melanoma cells generally do not produce IGF-1 and depend on exogenous sources for growth and survival^21, 37^. While little is known about the role of IGF-1 in the induction of tumor heterogeneity, therapy resistance in many cancers is mediated by IGF-1/IGF-1R signaling^4, 37^. Tumor stroma-derived cells, such as cancer-associated fibroblasts and macrophages, can release cytokines and growth factors including IGF-1^8^. We observed an increased expression of IGF-1 in melanoma lesions from patients treated with either BRAFi alone or BRAFi/MEKi combination therapies and increased IGF-1 correlated well with higher expression of FGFR-3 and CD20 (Fig. 7a). Immunostainings confirmed the expression of IGF-1 in CD20^+^/PAX5^+^ B cells and not in CD20^+^ melanoma cells (~2% of melanoma cells)^38^ (Fig. 7d; Supplementary Fig. 11a–c) and all patients with B-cell infiltration developed resistance to targeted therapies and tumor progression. Increase in CD20 and IGF-1 expressions was further confirmed in two cohorts of melanoma patients (GSE8401 and EGAS00001000992) who developed resistance to diverse therapies. These data are further supported by our clinical trial where the observation of anti-tumor activity through B-cell depletion alone in heavily pretreated, end-stage melanoma patients with high tumor load and, in several cases, disease progression under kinase/checkpoint inhibitor therapies is intriguing. Our data provide a mechanistic and clinical basis for the further clinical development of this therapeutic strategy as part of an alternative approach in cancer therapy, the interference with the pro-tumorigenic TME. Here, B-cell-targeted strategies in combination with established kinase or checkpoint inhibitor therapies are possible scenarios.

Our data also provide evidence for alternative strategies. The effect of TAB cells on melanoma heterogeneity and therapy resistance could be reversed by IGF-1 neutralization or FGFR-3 knockdown. Activation of FGFR-3 has been shown to reactivate Ras/MAPK signaling conferring resistance to BRAFi^39^ and combinations of pan-FGFR/-BRAF inhibitors have shown therapeutic benefits in mouse model^40^. Drugs targeting pan FGFR/VEGFR (Dovitinib, Lenvatinib (NCT00121680) and BGJ398 (NCT01820364)) are in clinical trials for testing their efficacy either alone or in combination with other therapy drugs.

## Methods

### Study design

Cell to cell interactions within the TME trigger release of pro-tumorigenic and pro-inflammatory cytokines and the objective of our study was to provide evidence for a cross-talk between TAB cells and tumor cells to induce therapy resistance of melanoma. For ex vivo experiments we used well-characterized (DNA finger printed) melanoma cell lines. B cells were obtained from tumor tissue extracts or peripheral blood from melanoma patients and healthy donors. Induction of IGF-1 in TAB cells and subsequent activation of FGFR-3 in melanoma cells contributed to kinase inhibitor therapy (BRAFi and MEKi) resistance. In addition, the expression of key molecules IGF-1, CD20 (B cells) and FGFR-3 was analyzed in matched patients’ tumors obtained before and on or post- (BRAFi or/and MEKi) therapy. For the ex vivo experiments, a minimum sample size of three technical and biological replicates was chosen. This leads to a power of 80%, with an alpha error of 5% to detect a 50% difference (estimated effect size of 1.8).

The details of the prospective, multicenter, open-label phase 2 pilot trial to assess the overall disease control rate of anti-CD20 therapy in therapy-resistant melanoma patients with unresectable stage III B (T1- 4a, N2b-c), III C or IV^41^ disease are available at https://clinicaltrials.gov/ct2/show/NCT01376713.The pre-planned primary endpoint disease control rate (*r*) was tested with a Simon two stage design comprising all patients that received at least one dose of the study drug. For all patients, the primary endpoint was available.

The optimal two-stage design with the one-sided type I error rate (the null hypothesis that *r* ≤ 0.05) bounded by 0.05 and a type II error rate (under the alternative that *r* ≥ 0.20) bounded by 0.2 had a pre-planned interim analysis of anti-CD20 therapy after a first stage sample size of 10 patients. As overall response evaluation at interim analysis revealed clear signs of clinical activity by anti-CD20 therapy but no confirmed CR or PR in overall response analysis and the rescue regimen (anti-CD20 therapy + dacarbazine) no additive anti-tumor activity (data not shown), the sponsor expected no further gain of information from the planned study design and decided to stop further recruitment into this trial.

### Reagents

Cisplatin and Paclitaxel were obtained commercially from Sigma-Aldrich (St Louis, MO), PLX4720 and PD0325901 were purchased from Selleckchem (Houston, TX). They were dissolved in H_2_O or DMSO and were stored at −20°C as 10 mM stocks.

Mouse anti-human IGF-1 neutralizing monoclonal antibody (clone Sm1.2 (IgG1))^42–44^ was obtained from Lifespan Biosciences (LS-C7504; Seattle, WA); goat anti-human FGF-2 neutralizing antibody was obtained from R&D Systems (AF-233-NA; Minneapolis, MN); rabbit anti-human FGFR-3 (ab10651), phospho-FGFR-3 (ab155960) and IGF-1 (ab9572) antibodies were obtained from Abcam Inc. (Cambridge, MA); mouse anti-human β-actin antibody was obtained from Sigma-Aldrich (A5316). Mouse anti-human CD20 was obtained from Dako (M0755; Carpinteria, CA). Mouse anti-human PAX5 (B-cell transcription factor; IgG1) was obtained from Leica Biosystems (PA0552; Buffalo Grove, IL). (Fluorochrome-conjugated antibodies for CD20 (11-0209), CD45 (12-9459), and CD146 (12-1469) were obtained from ebiosciences (San Diego, CA), CD133 (130-080-801) and CD271 (130-098-112) were obtained from Miltenyi Biotech (Auburn, CA) and FGFR-3 was obtained from R&D Systems (MAB766). CD271-dsRed promoter construct was obtained from Dr. Shi-Hua Li, Emory University, Atlanta, GA.

### Cell cultures and co-cultures

Human peripheral blood from normal healthy donors and human melanoma tissues were obtained in accordance with informed consent procedures approved by the Internal Review Boards of the Hospital of University of Pennsylvania, The Wistar Institute, the Medical University of Vienna, Massachusetts General Hospital Cancer Center, MD Anderson Cancer Center and Royal Prince Alfred Hospital, Sydney, Australia. Human melanoma cell lines have been previously described^23^ and they were cultured in DMEM medium/5% FBS. EBV-immortalized B-cell lines were established from freshly isolated peripheral blood lymphocytes cells (PBL) of normal healthy donors or tumor-infiltrating lymphocytes isolated from tumor tissues of metastatic melanoma patients^45^, cultured in RPMI1640 medium/10% FBS and used in co-culture experiments. All cell lines were tested for mycoplasma and short tandem repeat profile (DNA identity) before being used for any experiments. Tumor-infiltrating lymphocytes were isolated after mechanical mincing by sterile scalpels and enzymatic (Collagenase 1 mg/ml (Sigma-Aldrich) in RPMI1640 for 30 min at 37°C) disaggregation of tumor tissues and filtration through 70-μm cell strainer. Lymphocytes were enriched by negative selection by elimination of tumor cells using anti-CD146 coupled magnetic beads (Life Technologies, Grand Island, NY). B cells enriched further either by negative selection using anti-CD3 coupled magnetic beads or by EBV immortalization^46^. Most experiments were run with immortalized peripheral blood-derived normal and/or immortalized melanoma tumor-derived B cells (referred to as NB and TAB cells) and key experiments recapitulated with freshly isolated and non-immortalized B cells (referred to as “fresh NB” and “fresh TAB” cells). For tumor-B cells co-culture, exponentially growing melanoma cells (0.1–0.5 × 10^6^ cells) were seeded 1 day prior in the bottom chamber of six-well Transwell plates (0.4 μm pore size; Costar, Corning, NY) before the addition of an equal number of B cells in the top chamber. Melanoma cells or B cells were harvested at different time points for various assays. In some assays, IGF-1 or FGF-2 were neutralized in tumor-B cells co-culture by using anti-IGF-1(10 μg/ml) or anti-FGF-2 (1 μg/ml) antibodies. Cultures that received normal mouse or goat antibodies served as controls for comparisons.

### Cell viability and proliferation assay

Tumor cells (5 × 10^3^) were plated in complete medium in the presence/absence of DMSO control or PLX4720 or PD0325901 inhibitors. Cells were cultured for 72 h, at which time the medium received 1 × AlamarBlue (10% v/v; Life Technologies). Cells were allowed to reduce AlamarBlue for ~4 h and fluorescence was measured at 560/590 nm using a PerkinElmer Envision XciteMultilabel plate reader. Results are expressed as relative viability and are calculated as a proportion of fluorescence counts of tumor cells in the presence of drugs vs. tumor cells in the presence of vehicle control.

### ELISA assays

Supernatants from B cells or tumor-B-cell co-cultures were collected after 48–72 h for determining IGF-1 and FGF-2 production by using standard ELISA assays kits from R&D Systems (DY291-05; IGF-1) or from Peprotech (900-M08; FGF-2; Rocky Hill, NJ).

### Immunoblotting

For RTK phospho-protein array analysis, cell culture lysates were prepared and analyzed using a Pathscan RTK Signaling Antibody Array kit (Cell Signaling Technologies, 7949; Danvers, MA). Briefly, 100 µg lysate were loaded on an array membrane, incubated overnight at 4°C, and then washed with PBS/0.05% Tween, serially incubated for 1 h with the detection biotinylated antibody (150 μl/well; 1 × dilution) and DyLight-680-conjugated streptavidin (150 μl/well; 1 × dilution). Arrays were imaged using an Odyssey Infrared Imaging System and spot intensities were quantified using Image Studio software (Li-Cor, Lincoln, NE).

For western blots, protein cell extracts were obtained as previously described^4, 47^. Cell extracts (35 μg) were resolved on 10% polyacrylamide/SDS gels before being transferred onto polyvinylidene membranes (Millipore, Billerica, MA). Membranes were treated with primary antibodies at 1:1000 (anti-FGFR-3) or 1:2000 (anti-β actin) dilution followed by appropriate secondary anti-mouse (LICOR; 926-32220; IRDye 680LT) or anti-rabbit (LICOR; 926-32211; IRDye 800CW) antibodies (both at 1:10,000 dilution). Membranes were imaged and analyzed using an Odyssey Infrared Imaging System.

### Real-time PCR

Isolation of mRNA, cDNA synthesis and real-time qPCR were done as described previously^47^. In brief, qPCR was performed using an Applied Biosystems’ 7500 Fast Real-Time PCR System with Power SYBR Green PCR Master Mix (Life Technologies). The following primers, purchased from Integrated DNA Technologies (Coralville, IA), were used: IL-1α forward 5′-GAATGACGCCCTCAATCAAAGT and reverse 5′-TCATCTTGGGCAGTCACATACA; IL-1β forward 5′-AAACAGATGAAGTGCTCCTTCC AGG and reverse 5′-TGGAGAACACCACTTGTTGCTCCA; IGF-1 forward 5′-GCAATGGG AAAAATCAGCAG and reverse 5′-GAGGAGGACATGGTGTGCA; FGF2 forward 5′-GTGTGTGCTAACCGTTACCT and reverse 5′-GCTCTTAGCAGACATTGGAAG; FGFR-3 forward 5′-TCAGCTCCACAGCATCCC and reverse 5′-GTCCTTGGGGACGGAGC;PDGF-A forward 5′-CCCCTGCCCATTCGGAGGAAGAG and reverse 5′-TTGGCCACCTTGACGC T GCGGTG; PDFG-B forward 5′-GAT CCGCTCCTTTGATGATC and reverse 5′-GTCTCAC ACTTGCATGCCAG; VEGF-C forward 5′-TGCCGATGCATGTCTAAACT and reverse 5′-TGAACAGGTCTCTTCATCCAGC; CD20 (MS4A1) forward 5′-GTGAACCAGCTAATC CCTCTGA and reverse 5′-CAAGTTCCTGGAAGAAGGCA. Thermal cycler conditions were 95°C for 15 min and 40 cycles of 15 s at 95°C followed by 1 min at 60 °C. All experiments were performed in triplicate, and the mean value was used for the determination of mRNA levels. The relative quantification (RQ) and expression of each mRNA was calculated using the comparative *C*_T_ method according to the manufacturer (Applied Biosystems 7500 Software v2.0). All samples were normalized to an endogenous control, GAPDH.

### RNA FISH

RNA FISH was performed as described before with minor modifications^48^. Briefly, tumor cells grown in chamber slides (Nunclon, Denmark) after their co-culture with B cells were harvested, fixed and permeabilized, hybridized with the custom made FGFR-3 probes, then washed and imaged on a Nikon Ti-E equipped with appropriate filter sets^48^. The number of RNA in each cell was counted using custom image analysis scripts implemented in MATLAB.

### Gene expression array, RNA-seq and Kaplan–Meier survival analysis

Gene expression microarray data of pre- and post-treatment tumor biopsies of melanoma patients were downloaded from GEO under accession number GSE50509 (21 patients), and GSE8401 (51 patients). Illumina or Affymetrix chip data (GSE50509 and GSE8401) were normalized, background-corrected, and summarized using the R package “lumi” or “affy”^49, 50^.

RNA-seq data of The Cancer Genome Atlas (TCGA)-skin cutaneous melanoma (SKCM) patients (473 patients) were downloaded from TCGA Data Portal (https://tcga-data.nci.nih.gov/tcga/) and additional RNA-seq data were downloaded from EGA under accession number EGAS00001000992 (27 patients^51^). Data were summarized by feature Counts^52^ and then analyzed by using R package edgeR^50^. Tumors were first grouped into samples with a high and a low lymphocyte infiltrate based on expression levels of CD4, CD8, and CD20 (expression above the median of the whole group respectively, for clinical significance see Supplementary Fig. 2b). Then tumors with a high lymphocyte infiltrate were divided into two groups based on the expression levels of IGF-1 (above or below the median of the whole dataset). Differences in survival were analyzed using the log-rank test.

### Flow cytometry and immunostainings

Surface CD marker expression was determined by multi-color co-staining by FACS using an EPICS XL instrument (Beckman-Coulter), as described earlier^53^. FGFR-3 and phospho-FGFR-3 were detected with immunofluorescence as described earlier^54^. Briefly, cells were incubated with PBS/0.5% Triton X-100 for 5 min at RT followed by fixation in 4% paraformaldehyde (Sigma) for 10 min. Fixed cells were incubated with a rabbit anti-FGFR-3 or phospho-FGFR-3 antibody (both at 1:200 dilution) for 1 h at RT and were visualized by incubating the cells with Alexa-Fluor 488 conjugated goat anti-rabbit antibody (1:1,000 dilution). Nuclei were counterstained with DAPI (Life Technologies). Images were captured using Nikon TE-200 inverted microscope.

Immunostaining was performed essentially as previously described^55^. Briefly, slides were subjected to epitope-retrieval by incubation with target retrieval solution (Dako, Denmark) and were subsequently incubated 1 h at RT with a cocktail of mouse monoclonal antibodies against the three melanoma markers MCSP (melanoma associated chondroitin sulfate proteoglycan (commonly known as CSPG); originally developed in our laboratory^56^), β3 integrin (1:500 dilution; BD Biosciences, San Jose, CA; 555504) and HMB45 (1:500 dilution; Dako; M0634; all IgG1) followed by mouse monoclonal anti-CD20 (1:400 dilution; clone L26, Dako, IgG2A). For detection of FGFR-3 and IGF-1, slides were incubated with rabbit-anti humanFGFR-3 or IGF-1 polyclonal antibodies (both from Abcam and used at 1:1,000 dilution) at 4 °C overnight followed by incubation with mouse monoclonal antibodies against CD20 and MCSP (see above) for 1 h at RT. Melanoma marker, CD20, FGFR-3, and IGF-1 expression were visualized with Alexa488-conjugated goat anti-mouse IgG1 (A-21121), Alexa647-conjugated goat anti-mouse IgG2a (A-21241), and Alexa546-conjugated goat anti-rabbit IgG (A-11035) antibodies (all from Life Technologies and used at 1:1,000 dilution), respectively. Fixed tumor tissue sections from Massachusetts General Hospital and MD Anderson Cancer Center were sequentially stained for B-cell markers using mouse anti-human CD20 (Dako) or PAX-5 (1× ready to use dilution; Leica Biosystems) and rabbit anti-human IGF-1 (1:400 dilution; Abcam) as described earlier^57^. Binding of 2nd anti-mouse or anti-rabbit antibodies were visualized by brown (DAB) or red (Nova Red; Vector Laboratories, Burlingame, CA) chromogens^57^. Co-localization of IGF-1 in B cells was confirmed by using multi-spectral image analysis^57^. Nuclei were counterstained with DAPI. Respective isotype controls were used as a negative control. For tissue arrays, serial sections of melanoma specimens and normal controls were stained to assess reproducibility.

Tissue arrays were produced as single 0.5-mm punches taken from representative regions of paraffin-embedded materials as previously described^58^ and contained cores from human melanoma metastases representing subcutaneous and lymph node sites. Tissue arrays from paraffin blocks were produced after approval from the Ethics Committee of the Medical University of Vienna according to permission 405/2006 and extension 1930/2010. Slides were read by automated quantitative cytometry performed with a digital TissueFAXS imaging system and data analysis performed with Tissue Quest image analysis software (TissueGnostics, Vienna, Austria) essentially as previously described^59^. Briefly, each core was scanned using Observer Z1 microscope (Zeiss, Jena, Germany) and the mean relative fluorescence intensity (MFI) per cell for melanoma marker and/or CD20 expression plotted against the mean intensity of DAPI expression to determine the frequency of CD20-positive and melanoma marker-negative cells, respectively. Positive staining was automatically identified based on cutoff values set by engaging negative controls stained with isotype-matched control antibodies and the backward and forward gating tool of the software. CD20 + B cells were gated as CD20 + , triple melanoma marker-negative cells and results were displayed in scattergrams similar to flow cytometry. Criteria for inclusion of cores into the final analysis were: > 10% of cells positive for melanoma marker staining and, where more than one core from a tissue sample was analyzable, inclusion of the one with the highest frequency of CD20-positive cells. Using these criteria, of the 112 cores, 79 representing 48 different patients’ tissue samples were included in the final analysis.

### shRNA knockdown

Lentiviral particles from 5 individual clones from shRNA Target Sets NM_000142 targeting FGFR-3 and NM_021950 targeting MS4A1 (CD20) were used to infect melanoma cell lines. Twenty-four to 48 h post-infection, 2.5 μg/ml puromycin was added to select for infected cells and the infection efficiency was ~95%. Resistant cells were maintained with 1 μg/ml puromycin. As a control, a non-targeting shRNA was used for infection.

### Pilot trial of CD20 immunotargeting in stage IV melanomas

A small, prospective, multicenter, open-label, interventional pilot-trial with the anti-CD20 antibody Ofatumumab was conducted in patients with therapy-resistant advanced, end-stage metastatic cutaneous melanoma (study medication provided by GSK; ClinicalTrials.gov number, NCT01376713). The study protocol was approved by the institutional ethics committees of the trial centers and the study was managed by the Academic Clinical Studies Support Office of the Medical University of Vienna in accordance with the Declaration of Helsinki and International Conference on Harmonization Guidelines for Good Clinical Practice. Informed consent was obtained from all patients; an independent Data Safety and Monitoring Committee took care of additional safety oversight.

We included 10 (cutaneous) melanoma patients who (i) had received the diagnosis of measurable, non-resectable stage IV melanoma (AJCC 2009)^41^, (ii) had experienced progressive disease under kinase and/or checkpoint inhibitors or, who could not be considered for those therapies (e.g., with tumors not carrying the respective mutational profile, not eligible due to contraindications or refusing this kind of therapy for any reason), other standard treatments and (iii) had an ECOG (Eastern Cooperative Oncology Group) performance status of 0-1, adequate organ functions, and a life expectancy of at least 3 months. Exclusion criteria included patients with active, untreated brain metastasis, immunoglobulin-deficiency, a clinically significant cardiac or cerebrovascular disease, a hepatitis B or C infection, and a human immunodeficiency virus infection.

Ofatumumab was administered intravenously at a dose of 1,000 mg weekly for 8 weeks and every 4 weeks for another 16 weeks. Tumor imaging (magnetic resonance imaging (MRI), computed tomography (CT) or routine whole body ^(18)^F-fluorodeoxyglucose positron emission tomographic (FDG-PET)-CT scans) was performed at week 4 (screening for rapid disease progression), 8, 16, and 24 (all ± 1 week). Scans were read by two independent study radiologists including a nuclear medicine physician, who decided on continuation of therapy. In case of disease progression, patients had the opportunity to switch to a rescue arm to receive intravenously at least 3 cycles of a combination therapy with dacarbazine (1,000 mg/m^2^) every 4 weeks (Supplementary Fig. 12). The pre-planned primary end point of this study was to evaluate the overall disease control rate (either CR, PR, or SD),pre-planned secondary end points included assessment of progression-free survival (see duration of SD, results section) and safety outcome according to NCI-CTC and -CTCAE v.4.03 criteria (not shown).

### Statistics

Kaplan–Meier survival analysis, Student’s *t*-test and Spearman’s rank correlation coefficient were used. A *p*-value of <0.05 is considered significant. As software tools, R statistical package, Stata 13, Microsoft Excel and MATLAB were used.

### Data availability

Data on the phase 2 pilot trial are available at https://clinicaltrials.gov/ct2/show/NCT01376713.

Microarray data of pre- and post-treatment tumor biopsies of melanoma patients are available from GEO under accession number GSE50509 and GSE8401. Illumina or Affymetrix chip data are available under accession number GSE50509 and GSE8401. The Cancer Genome Atlas (TCGA)-skin cutaneous melanoma (SKCM) patients are available at https://tcga-data.nci.nih.gov/tcga/ and RNA-seq data are available under accession number EGAS00001000992.

## Electronic supplementary material

Supplementary Information
