# Supplementary material for: Tumor-associated B-cells induce tumor heterogeneity and therapy resistance
Source: Nat Commun. 2017 Sep 19;8:607. doi: 10.1038/s41467-017-00452-4 (PMC5605714; doi:10.1038/s41467-017-00452-4)
Supplement: Supplementary file 1 — Supplementary Information [file 41467_2017_452_MOESM1_ESM.pdf]

Description of Supplementary Files

File Name: Supplementary Information
Description: Supplementary Figures and Supplementary Tables

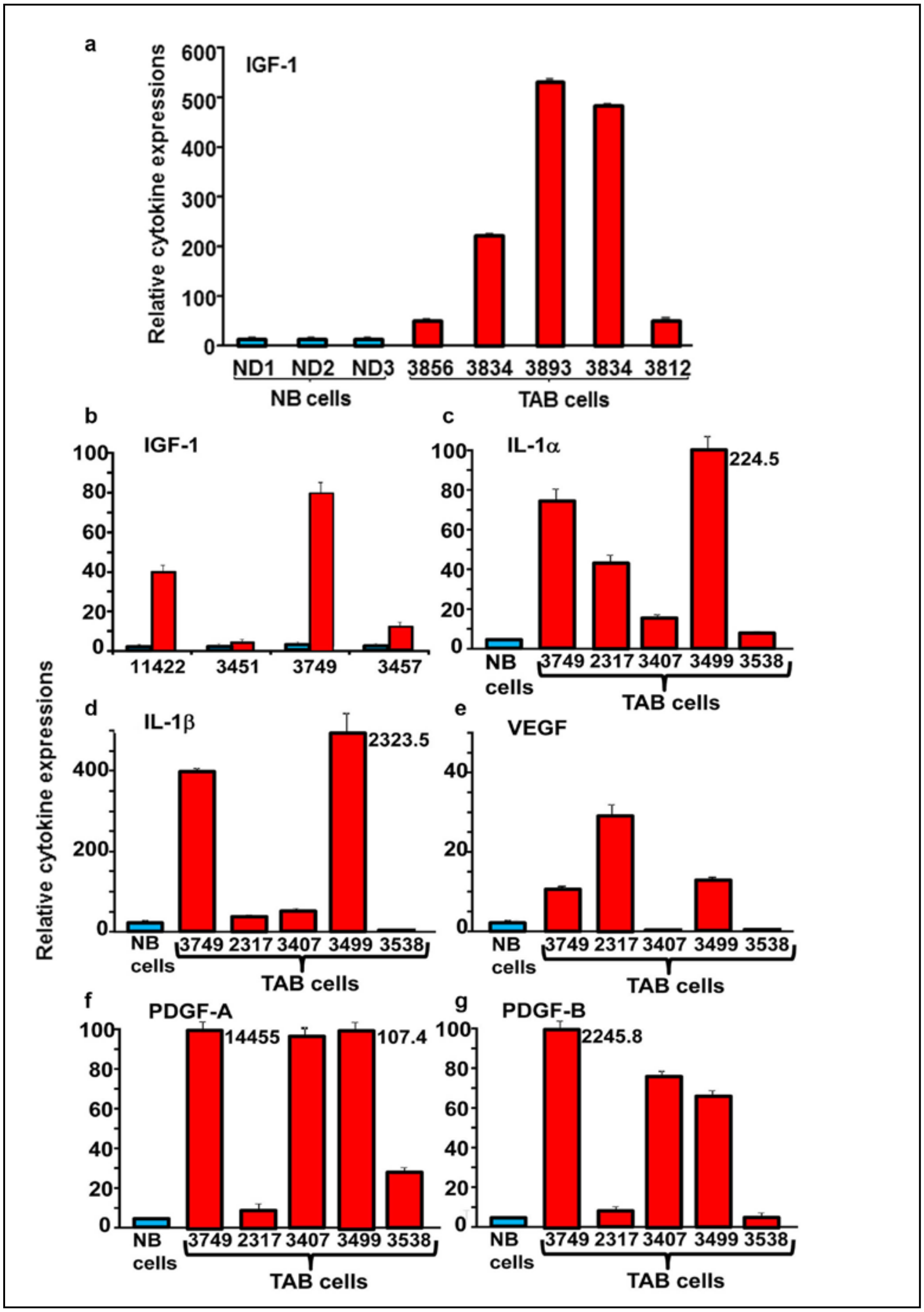

Supplementary Figure 1. Increased inflammatory cytokine mRNA transcript expression in tumor-associated B (TAB) cells. (**a**) Fresh TAB-cells directly isolated from patients' melanoma tissues (**TAB cells**; **red bars**) show increased expression of IGF-1 when compared to fresh NB-cells from peripheral blood (**blue bars**). (**b**) Immortalized TAB-cells from melanoma tissues (**TAB cells**; **red bars**) show increased expression

of IGF-1 when compared to immortalized NB-cells from same patients' peripheral blood (**blue bars**). (**c-g**) Immortalized B-cells from melanoma tissues (**TAB cells**; **red bars**) show increased expression of IL-1α (**c**), IL-1β (**d**), VEGF (**e**), PDGF-A (**f**) and PDGF-B (**g**) when compared to immortalized NB cells from healthy volunteers (**blue bars**). mRNA transcripts were determined by real time qPCR as in Fig. 1D with levels indicated as RQ values normalized to an endogenous GAPDH control and relative to normal B (NB) cells from peripheral blood. Bars represent mean $\pm$ SE of replicate samples. Results are representative of 3 independent experiments for each sample.

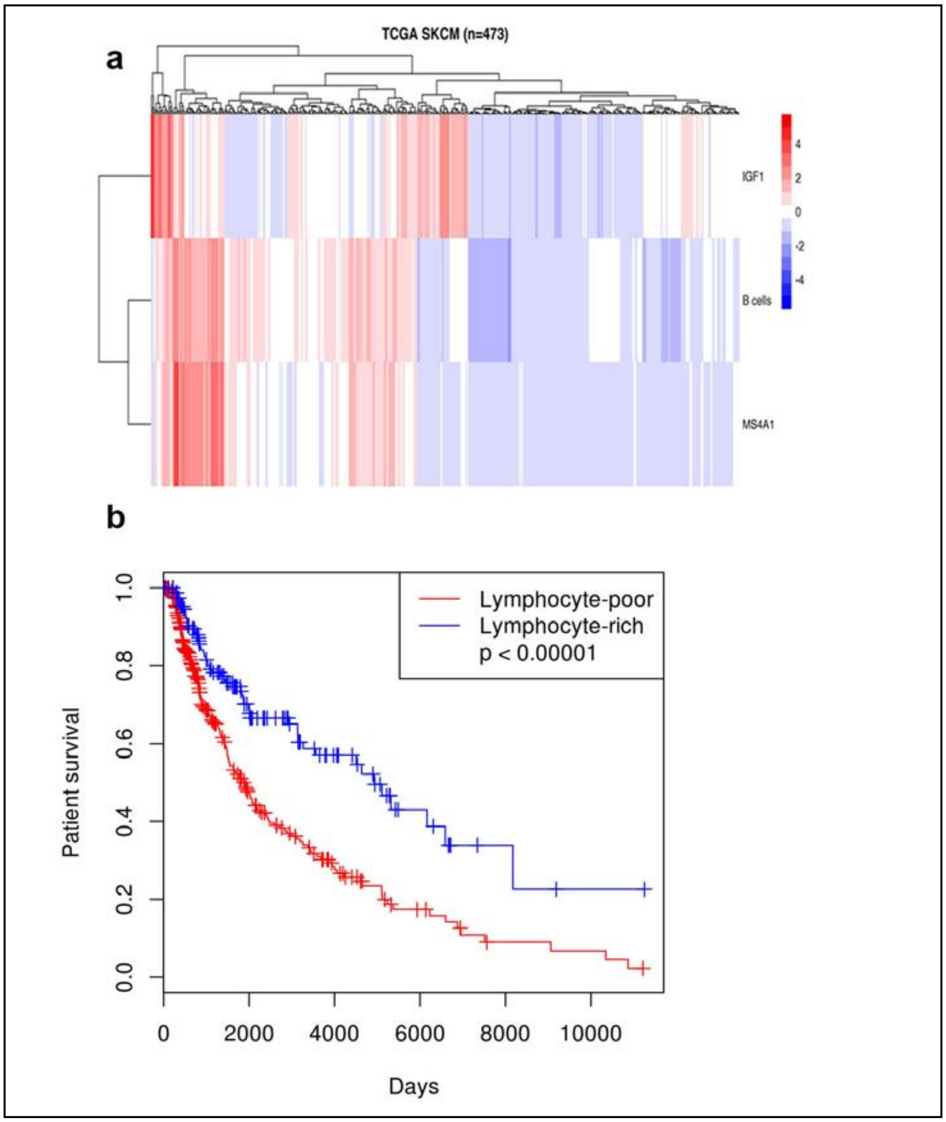

Supplementary Figure 2. TCGA melanoma patients' data set analyses; **a lymphocyte-expression signature with clinical significance in melanomas. (a)** The heat map of the TCGA data set (n=473) showing clustering of *IGF1* with B cells and *MS4A1* genes. **(b)** Kaplan-Meier survival curves of melanoma patients who were divided into high and low expression for lymphocyte markers CD4, CD8, and CD20 (by median, see Methods). Log-rank test shows a favorable OS in melanoma patients with tumors containing a high lymphocyte infiltrate.

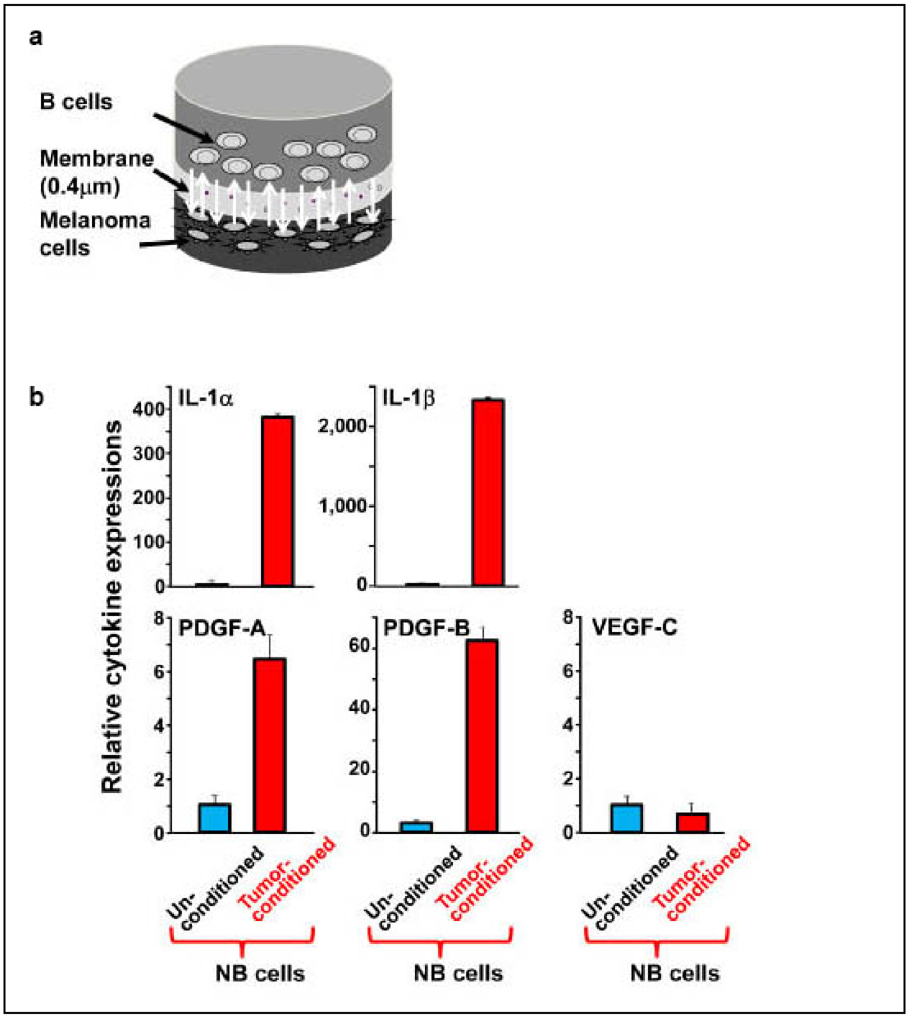

Supplementary Figure 3. (a) Culture schema: Melanoma cells (bottom chamber) were co-cultured with B-cells (either NB or TAB cells; top chamber) in a transwell plate (separated with a membrane [0.4 μM] pore size that allows only soluble growth factor permeability) for 5-14 days. B-cells or tumor cells were then harvested and used for various assays. **(b) Tumor-conditioned normal B-cells show increased inflammatory cytokine expression.** NB-cells co-cultured with tumor (WM3749; tumor-conditioned; **red bars**) show increased expression of cytokines including IL-1α, IL-1β, PDGF-A and PDGF-B compared to unconditioned NB-cells from the same healthy volunteer (**blue bars**). NB-cells were harvested after 14 days of co-culture and analyzed by qPCR as described in Fig. 1D. Results are representative of 2 independent experiments.

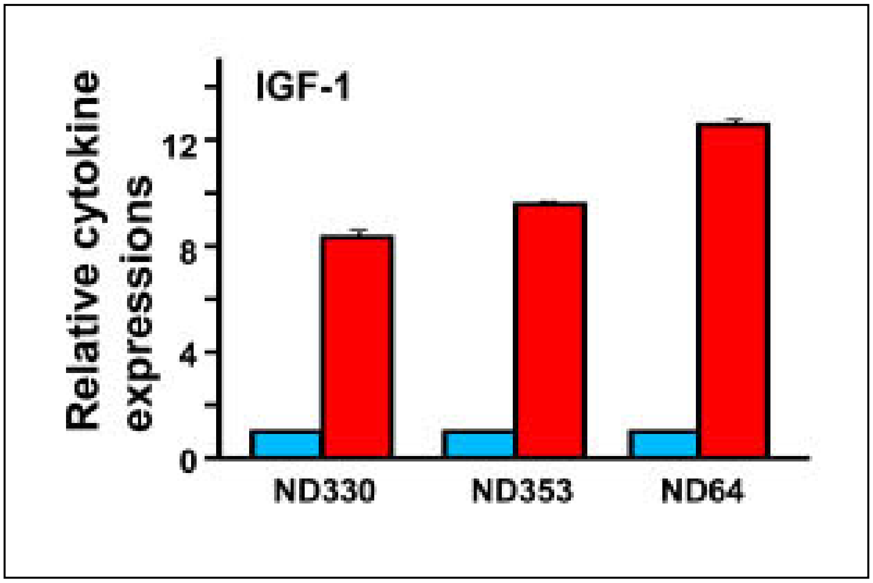

Supplementary Figure 4. Tumor-conditioned fresh NB-cells show increased IGF-1 expression. Non-immortalized B-cells isolated by negative selection by using anti-CD3/anti-CD146 coupled beads from peripheral blood and co-cultured with melanoma tumor cells (WM3749; tumor-conditioned; **red bars**) show increased expression of IGF-1 when compared to unconditioned fresh NB-cells from the same healthy volunteer (**blue bars**). As non-immortalized B-cells were short lived they were harvested based on cell viability (>90%; days 7-10 and analyzed by qPCR as described in Fig. 1D.

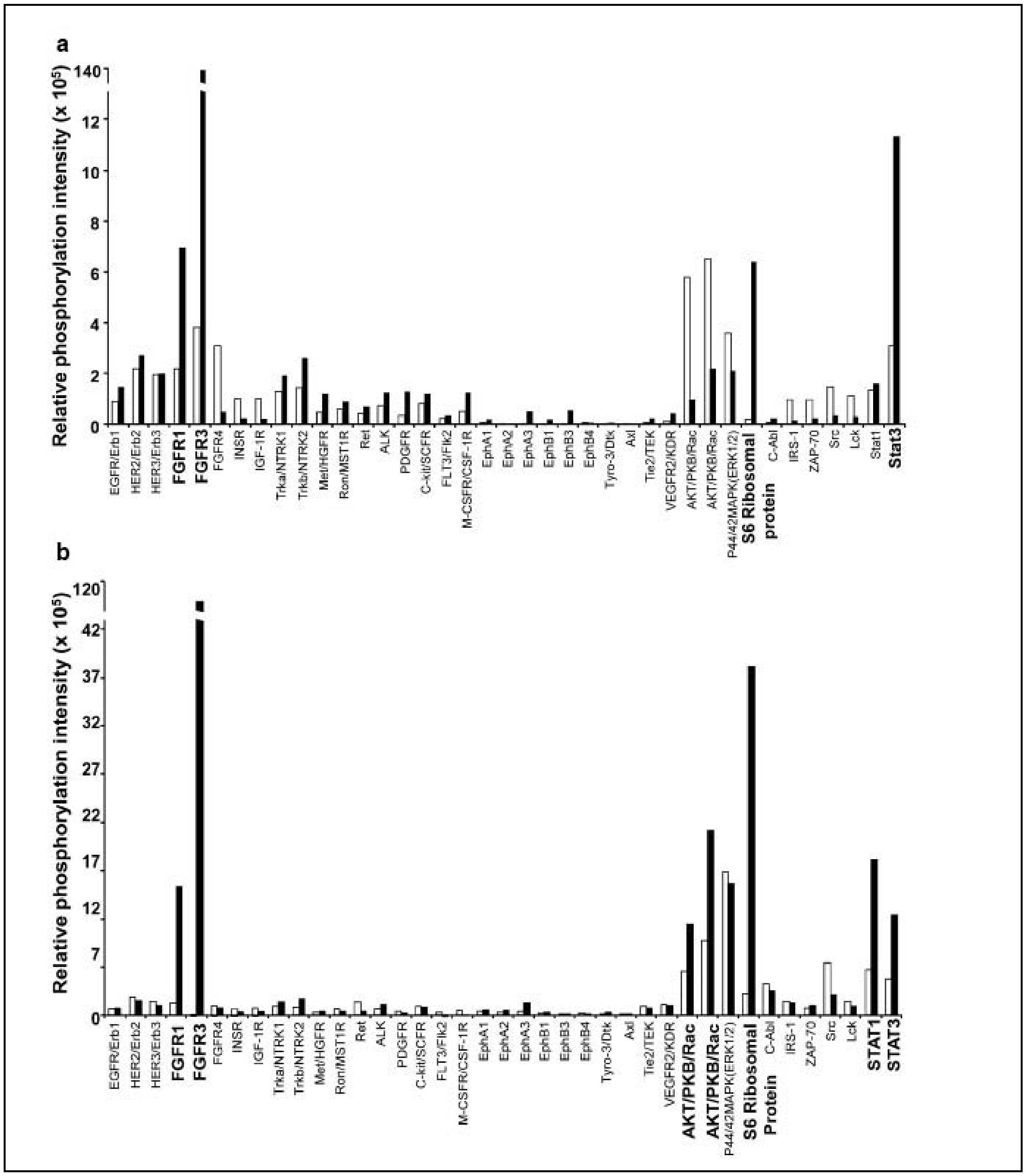

Supplementary Figure 5. Phospho-RTK array analyses of B-cells co-cultured with melanomas show increased phospho-signaling of FGFR-1, FGFR-3, S6 Ribosomal protein and STAT3. (a) Cell lysates from tumor-conditioned NB-cells (**black bars**) show increased phospho-signaling of FGFR-1, FGFR-3, S6 Ribosomal protein and STAT3 compared to unconditioned NB-cells (**open bars**) as determined by phospho-RTK array analysis at 48h. (**b**) As in (A), cell lysates of tumor-conditioned TAB-cells (black bars) show increased phospho-signaling of FGFR-1, FGFR-3, AKT/PKB/Rac, S6 Ribosomal protein, STAT1 and STAT3 compared to control unconditioned TAB-cells (**open bars**) at 48h.

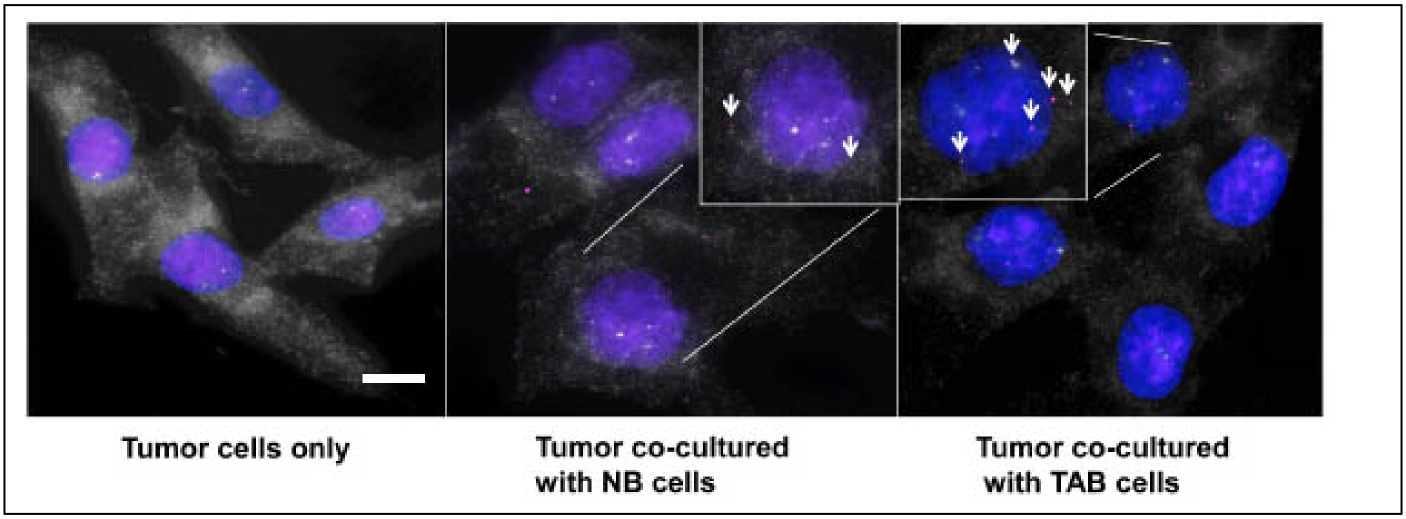

Supplementary Figure 6. Melanoma cells after co-culture with B-cells showed increased FGFR-3 expression. Melanoma cells (WM3749) grown in chamber slides in the presence of TAB-cells (**right panel**) show increased FGFR-3 transcripts (**white arrows**) at the single cell level as detected by custom made FGFR-3 fluorescent probes when compared to tumor cells either cultured alone (**left panel**) or cultured in the presence of NB-cells (**middle panel**). Nuclei are stained with DAPI. Scale bars: 10 µm.

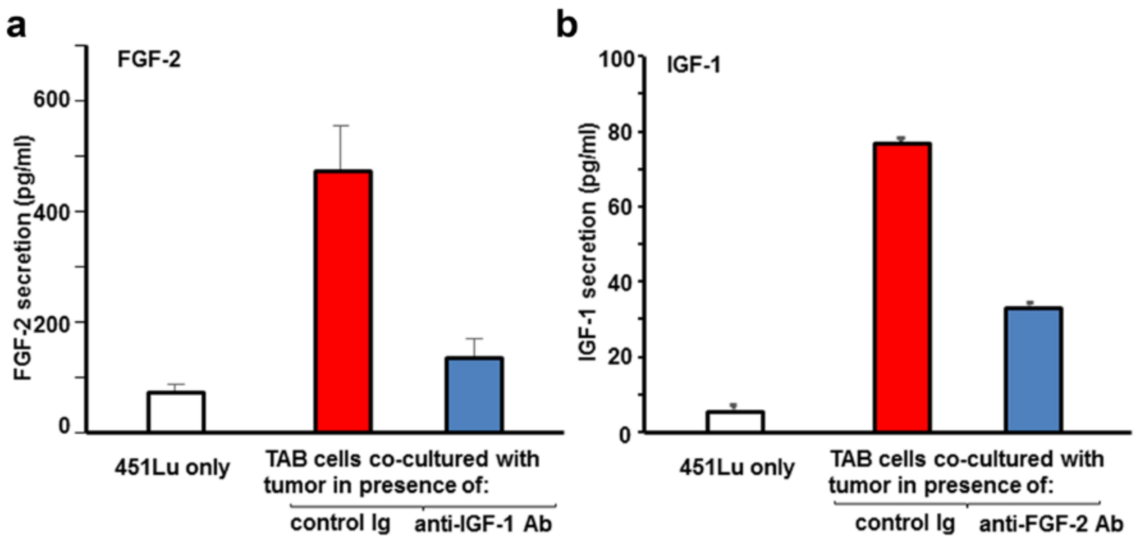

Supplementary Figure 7. Inhibition of FGF-2 and IGF-1 in melanoma-TAB cell co-cultures in presence of anti-IGF-1 and anti-FGF-2 neutralizing antibodies. (a) Melanoma cells (451Lu) co-cultured (72h) with TAB-cells in the presence of an anti-IGF-1 neutralizing antibody (10 μg/ml) show decreased FGF-2 secretion in culture supernatants (**blue bar**) when compared to cultures in presence of control antibody (**red bar**). **(b)** TAB-cells co-cultured (72h) with melanoma cells (451Lu) in the presence of an anti-FGF-2 neutralizing antibody (1 μg/ml) show decreased IGF-1 secretion in culture supernatants (**blue bar**) when compared to cultures in presence of control antibody (**red bar**). FGF-2 and IGF-1 protein expressions was determined by using an ELISA kit. Bars represent mean ± SE of triplicate samples.

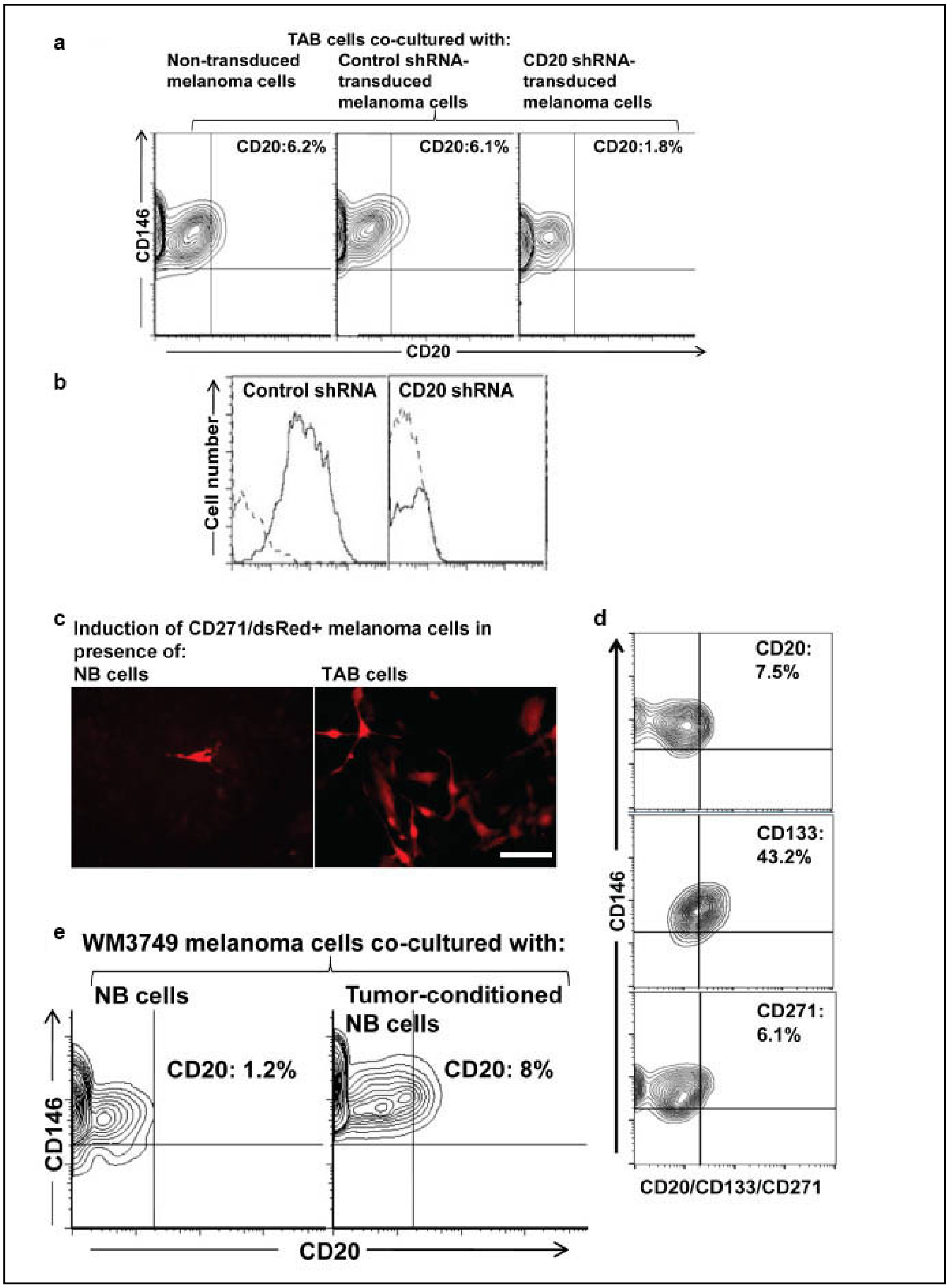

Supplementary Figure 8. Induction of CD20, CD133 or CD271 in melanoma cells after co-culture with B-cells. (a) Induction of CD20 in melanoma cells by co-cultured TAB-cells (day 6; see Fig. 4B) is blocked when melanoma cells (WM3749) are transduced with CD20 shRNA. Induction of CD20 in melanoma cells is not blocked in non-transduced melanoma cells or melanoma cells transduced with control shRNA, FACS assay, melanoma cell staining for CD146 (MCAM) **(b)** Validation of the CD20 shRNA clone: Minimal

CD20 expression of normal B-cells after transduction with CD20 shRNA, FACS assay. **(c)** Induction of CD271 upon co-culture with TAB-cells in melanoma cells transduced with a CD271/dsRed promoter construct (right). Minimal induction of CD271 was seen in melanoma cells co-cultured with un-conditioned NB-cells (left). Scale bars: 100 μm. (**d**) Unlike neutralizing anti-IGF-1 antibody, control mouse Ig is unable to block CD20, CD133 or CD271 expression in melanoma cells upon co-culture with TAB-cells (see Fig. 4B). **(e)** Tumor-conditioned NB-cells (see Fig. 2) up-regulated CD20 expression in melanoma cells after co-culture (day 6) as determined by FACS assay. Unconditioned normal B-cells had a minimal effect on CD20 expression in tumor cells.

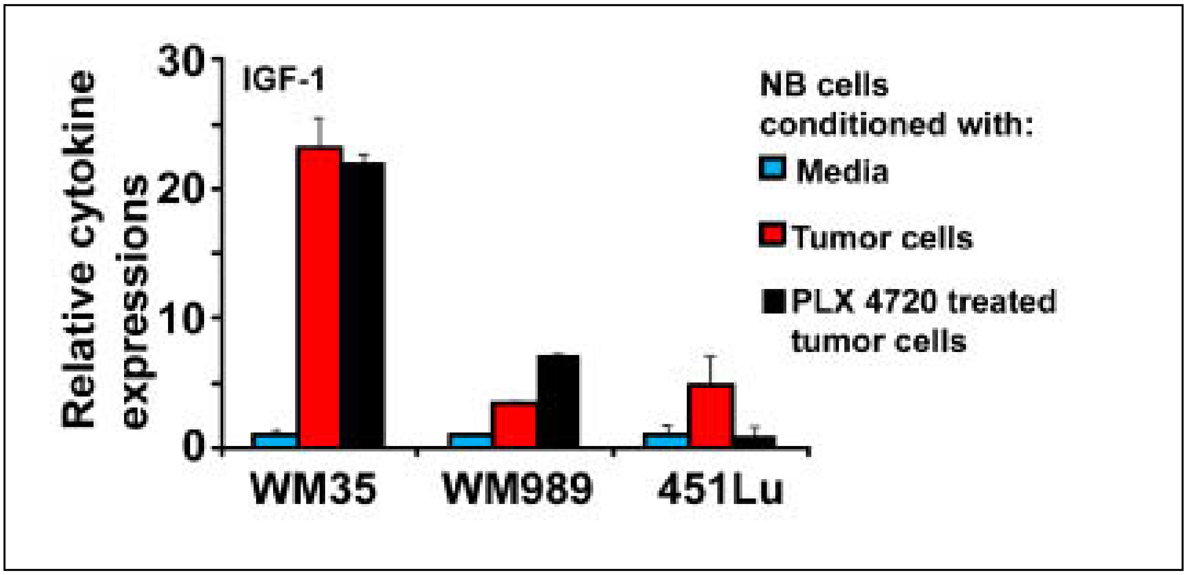

Supplementary Figure 9. BRAFi-treated tumor cells can preserve their ability to induce IGF-1 in B-cells. NB-cells were co-cultured with either medium alone (**blue bars**) or with melanoma cells that had received no treatment (**red bars**) or treatment with BRAF inhibitor (1μM PLX4720 for 72h; **black bars**). NB cells were harvested and analyzed for IGF-1 expression by qPCR as described in Fig. 1D. Bars represent mean $\pm$ SE of duplicate samples. Results are representative of 2 independent experiments.

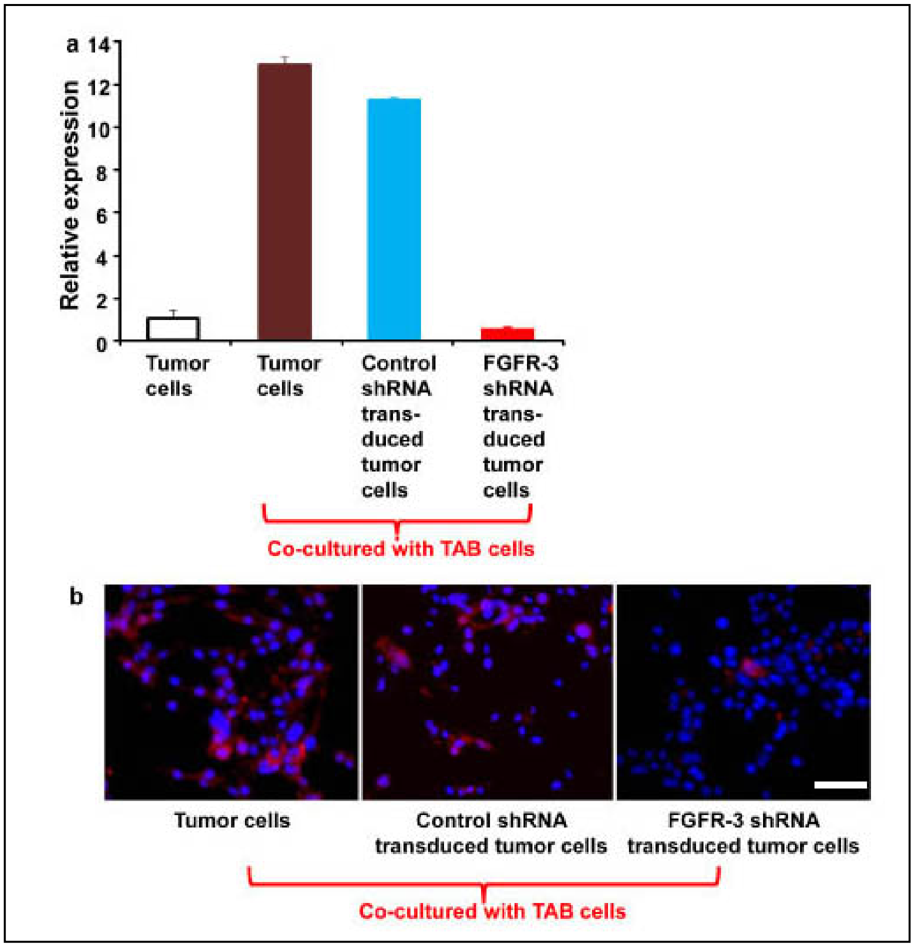

Supplementary Figure 10. Validation of FGFR-3 shRNA clone. (**a**) Melanoma cells (451Lu) transduced with FGFR-3 shRNA show minimal expression of FGFR-3 after co-culture (48h) with TAB-cells as compared to un-transduced or control shRNA-transduced co-cultured melanoma cells as determined by qPCR (Bars represent mean $\pm$ SE of duplicate samples) or (**b**) by immunofluorescence assay as in Fig 3C. Scale bars: 100 μm.

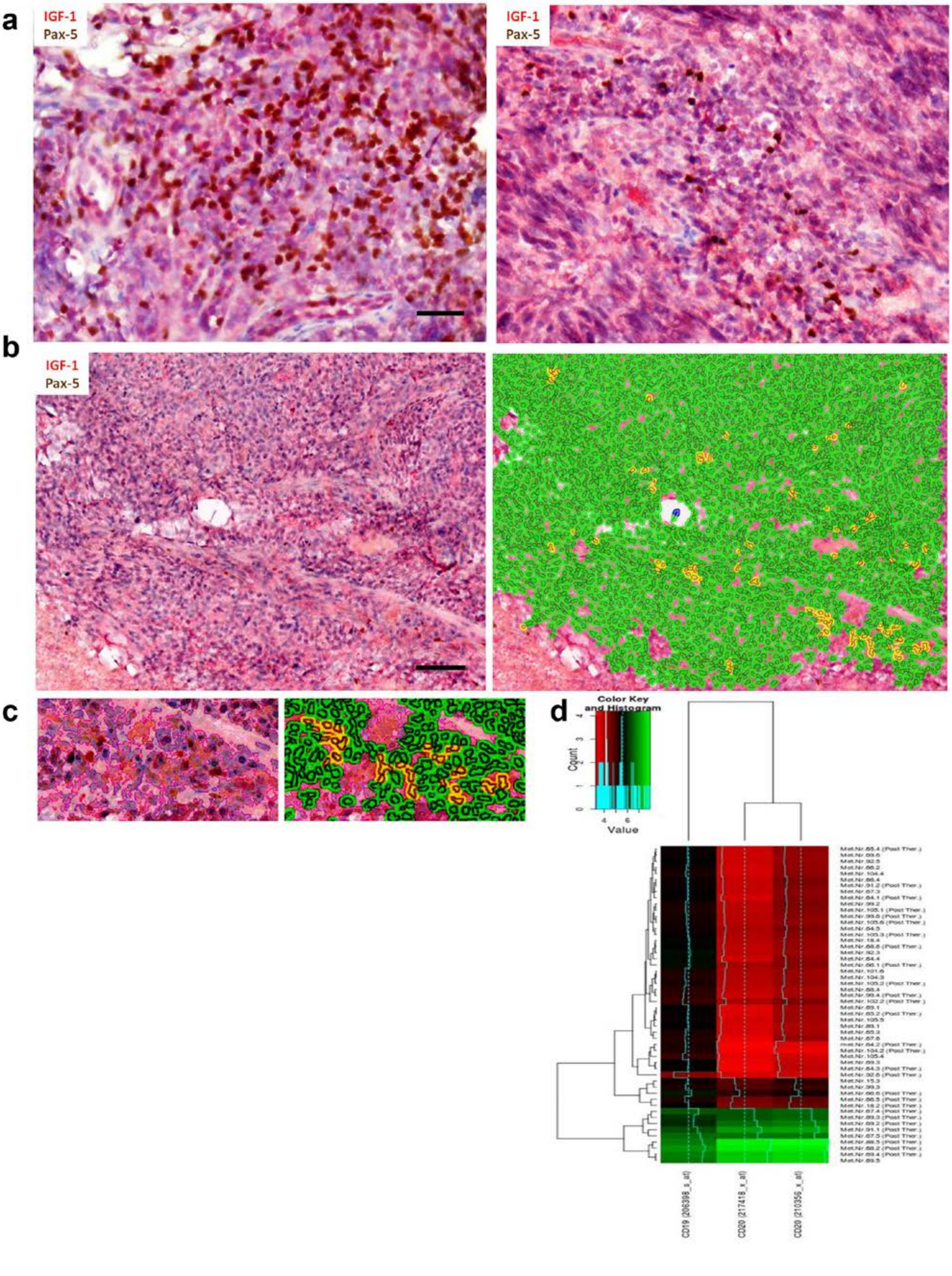
a
IGF-1
Pax-5
IGF-1
Pax-5
b
IGF-1
Pax-5
c
d
Color Key and Histogram
Count
Value
CD19 (206398_s_at)
CD20 (217418_x_at)
CD20 (210356_x_at)

Supplementary Figure 11. Increased presence of PAX5$^+$ B-cells co-stained with IGF-1 in tumor sections obtained from patients undergoing treatment with a combination of BRAF and MEK inhibitors. (a-c) Representative immunostaining of three patients undergoing treatment showing co-staining of IGF-1 (**red**) and PAX5 (B-cell transcription factor nuclear staining; **dark brown;** 400x magnification for top panels, scale bars: 25 μm. **(a)** and 100x magnification for bottom panels, scale bars: 100 μm. (**b**). Multi-spectral analysis confirming the co-localization of IGF-1 and PAX5$^+$ B-cells is shown in the bottom right panel (**yellow**). None of the pre-therapy sections showed PAX5$^+$ B-cells (data not shown). **(c)** Magnified view of the co-staining is shown on the middle left and multi-spectral analysis confirming the co-localization of IGF-1 and CD20+ B-cells is shown in **middle right panel** (**yellow**). Co-localization of IGF-1 (**red**) and Pax5+ B-cells (**dark brown**) is shown in the lower left panel, the corresponding multi-spectral analysis confirming their co-localization (**yellow**) on the lower right. **(d) Increased *MS4A1* and CD19 expression in a subgroup of tumor biopsies.** Increased *MS4A1* (encoding CD20) along with CD19 gene expression in tumor biopsies of melanoma patients (n=52) who were untreated or had developed resistance to diverse type of therapies including chemo-, radiation- and immune-therapies (Post Treat.). Note enrichment of CD20/CD19 high expressor samples for therapy-resistant samples (Post Treat.)**.** Gene expression analysis was performed using GEO dataset (GSE8401).

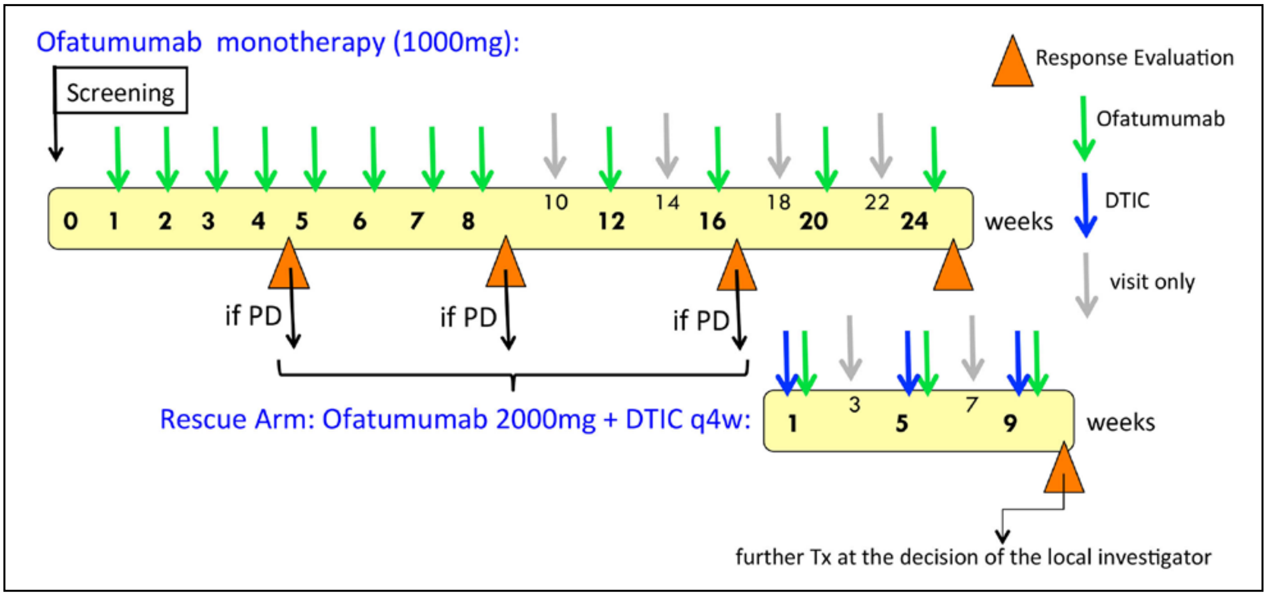

Supplementary Figure 12. Clinical pilot trial design. Ten eligible patients were treated with ofatumumab (1000 mg) alone. Tumor imaging was performed at wk 4 (screening for rapid disease progression), 8, 16 and 24. Follow-up started at wk 24. Note that all clinical data are reported for treatment with ofatumumab alone. Rescue arm: if ofatumumab alone at the 1000 mg dose was well tolerated, patients with disease progression had the opportunity to receive at least 3 cycles of ofatumumab q4w at an increased dose of 2000 mg in combination with dacarbazine (DTIC).

Supplementary Table 1

B-cell signature genes.

Cell Type	B-cell signature genes
B cells	*ABCB4, BACH2, BCL11A, BLK, BLNK, CCR9, CD19, CD72, COCH, CR2, DTNB, FCRL2, GLDC, GNG7, HLA-DOB, HLA-DQA1, IGHA1, IGHG1 IGHM, IGKC, IGL, KIAA0125, MEF2C, MICAL3, MS4A1, OSBPL10, PNOC, QRSL1, SCN3A, SLC15A2, SPIB, TCL1A, TNFRSF17*

Supplementary Table 2

Patient, treatment and therapy response.

Patient number	Treatment	Response	Time to progression (months)
2	Vemurafenib	PR (-60.5%)	8.5
4	Vemurafenib	PR (-56%)	3.5
5	Vemurafenib	SD (-27%)	6.5
6	Dabrafenib +trametinib	PR (-59.9%)	21
7	Dabrafenib +trametinib	CR (100%)	17, ongoing 47 months
9	Dabrafenib +trametinib	PR (-45%)	7
10	Dabrafenib +trametinib	SD (-13%)	3
11	Dabrafenib +trametinib	PR (-80%)	10
12	Dabrafenib +trametinib	PR (-88.9%)	12, stopped at 20 months
13	Dabrafenib +trametinib	PR (-57.9%)	9, stroke
16	Dabrafenib +trametinib	SD (-19.5%)	11
20	Vemurafenib	PR (-57.9%)	5
24	Dabrafenib +trametinib	SD (-19.5%)	11
25	Dabrafenib +trametinib	SD (-19.5%)	11
34	LGX8181+MEK162	PR (-48.6%)	Stopped after 14 months, PD at 15 months
35	LGX8181+MEK162	SD (-22.8%)	Stopped after 7 months, PD at 10 months
38	Vemurafenib	SD (-24.9%)	4.3
40	Vemurafenib	SD (-19.5%)	Stopped after 7 months, PD at 10 months
42	LGX8181 +MEK162	SD (-19.5%)	13
43	Vemurafenib	PR (-57.9%)	13.4

All patients are BRAFV600E mutation positive.
CR: complete response, PR: partial response, PD: progressive disease, SD: Stable disease.

Supplementary Table 3.
The percentage of tumor area that shows CD20+ B cell infiltration in IHC staining of melanoma tissues from patients with increased FGFR3 expression in progression biopsies (from GEO dataset GSE50509)

Patient number	Disease status	Peritumoral CD20		Intratumoral CD20	
		% area[a]	Density[a]	% area[a]	Density[a]
3	Pre	5	3	5	3
	Progression 1[b]	2	2	2	2
	Progression 2	2	2	2	2
7	EDT[c]	5	1	5	1
	Progression	5	2	1	1
10	Pre	No tumor	-	1	1
	EDT	1	1	1	1
	Progression	5	2	1	1
11	Pre	20	1	30	2
	Progression 1	NA[d]	NA	5	2
	Progression 2	NA	NA	1	1
14	Pre	NA	NA	1	1
	Progression	0	0	1	1
18	Pre	20	2	2	2
	Progression 1	3	2	0	0
	Progression 2	1	1	1	1
21	Pre	5	2	2	2
	Progression	5	2	20	1

[a] Score from 0= no lymphocytes, 1= 1-10/high magnification field (HPF), 2=11-50 HPF, 3$\geq$50 HPF.
[b]Melanoma patients resistant to BRAF inhibitor therapy drug Dabrafenib.
[c]EDT: early during treatment.
[d]NA: Not assessable.

Supplementary Table 4.

Baseline patients' demographic and clinical characteristics

Patient	Age (years), gender	ECOG performance status	TNM (AJCC 2009)	Metastasis Number of sites	Metastasis Sites	Prior therapies
1	35, m	0	M1c	5	Skin, subcutaneous, lymph node, lung, liver	Surgery, IFN, ipilimumab
2	48, m	1	M1b	4	Skin, subcutaneous, lymph node, lung	Surgery, IFN, dacarbazine, ipilimumab
3	72, m	0	M1c	4	Subcutaneous, lymph node, lung, liver	Surgery, dacarbazine
4	53, f	0	M1c	5	Soft tissue, lymph node, lung, liver, bone	Surgery, vemurafenib, ipilimumab
5	68, m	0	M1c	2	Skin, soft tissue	Surgery, dacarbazine, fotemustine, radiotherapy, carboplatin/paclitaxel, ipilimumab
6	51, m	0	M1b	4	Skin, subcutaneous, lymph node, lung	Surgery
7	32, f	0	M1a	3	Skin, subcutaneous, lymph node	Surgery, paclitaxel, cisplatin, vemurafenib, ipilimumab
8	66, m	0	M1c	3	Lung, liver, bone	Surgery, IFN, IL-2, ipilimumab, radiotherapy, fotemustine, carboplatin/paclitaxel
9	71, m	0	M1c	4	Skin, subcutaneous, lymph node, liver	Surgery, IFN
10	72, m	0	M1c	4	Lymph node, lung, liver, bone	Surgery, IFN, dacarbazine, IL-2, ipilimumab

Abbrev.: M1a, distant skin, subcutaneous, or nodal metastases; M1b, lung metastases; M1c, all other visceral metastases.

Supplementary Table 5.

Tumor Measurements (measurable disease, all patients)

**Best Response** (ITT analysis)	Ofatumumab (n=10, %)
**Complete response (CR)**	0
**Partial response (PR, confirmed)**	1 (10)
**Stable Disease (SD)**	5 (50)
**with Tumor mass decrease by RECIST v.1.1/irRC**	4/5 (80)
**Disease control (CR+PR+SD)**	6 (60)
**Progressive disease (PD)**	4 (40)
**with Induction of necrotic tumor mass or reduction of SUV**	2/4 (50)
